# Mathematical modeling in autoimmune diseases: from theory to clinical application

**DOI:** 10.3389/fimmu.2024.1371620

**Published:** 2024-03-14

**Authors:** Yaroslav Ugolkov, Antonina Nikitich, Cristina Leon, Gabriel Helmlinger, Kirill Peskov, Victor Sokolov, Alina Volkova

**Affiliations:** ^1^ Research Center of Model-Informed Drug Development, Ivan Mikhaylovich (I.M.) Sechenov First Moscow State Medical University, Moscow, Russia; ^2^ Marchuk Institute of Numerical Mathematics of the Russian Academy of Sciences (RAS), Moscow, Russia; ^3^ Faculty of Bioengineering and Bioinformatics, Lomonosov Moscow State University, Moscow, Russia; ^4^ Modeling and Simulation Decisions FZ - LLC, Dubai, United Arab Emirates; ^5^ Biorchestra Co., Ltd., Cambridge, MA, United States; ^6^ Sirius University of Science and Technology, Sirius, Russia

**Keywords:** autoimmune diseases, mathematical modeling, quantitative systems pharmacology, model-informed drug development, immune system modeling

## Abstract

The research & development (R&D) of novel therapeutic agents for the treatment of autoimmune diseases is challenged by highly complex pathogenesis and multiple etiologies of these conditions. The number of targeted therapies available on the market is limited, whereas the prevalence of autoimmune conditions in the global population continues to rise. Mathematical modeling of biological systems is an essential tool which may be applied in support of decision-making across R&D drug programs to improve the probability of success in the development of novel medicines. Over the past decades, multiple models of autoimmune diseases have been developed. Models differ in the spectra of quantitative data used in their development and mathematical methods, as well as in the level of “mechanistic granularity” chosen to describe the underlying biology. Yet, all models strive towards the same goal: to quantitatively describe various aspects of the immune response. The aim of this review was to conduct a systematic review and analysis of mathematical models of autoimmune diseases focused on the mechanistic description of the immune system, to consolidate existing quantitative knowledge on autoimmune processes, and to outline potential directions of interest for future model-based analyses. Following a systematic literature review, 38 models describing the onset, progression, and/or the effect of treatment in 13 systemic and organ-specific autoimmune conditions were identified, most models developed for inflammatory bowel disease, multiple sclerosis, and lupus (5 models each). ≥70% of the models were developed as nonlinear systems of ordinary differential equations, others – as partial differential equations, integro-differential equations, Boolean networks, or probabilistic models. Despite covering a relatively wide range of diseases, most models described the same components of the immune system, such as T-cell response, cytokine influence, or the involvement of macrophages in autoimmune processes. All models were thoroughly analyzed with an emphasis on assumptions, limitations, and their potential applications in the development of novel medicines.

## 1 Introduction

Autoimmune diseases (ADs) are a group of diverse disorders that occur widely and affect approximately 12.5% of the global population, with a greater prevalence among childbearing women (1). A landmark feature in ADs can be found in immune disturbances causing autoreactivity of lymphocytes against normal cells of the organism (2). Even though all ADs share a common pathophysiological basis of development, their clinical manifestations could vary from mild abnormalities in laboratory measurements to life-threatening conditions such as organ failure following serious tissue damage (3). Depending on the origin of their manifestation, ADs could be restricted to a single organ (organ-specific ADs) such as the thyroid gland in Hashimoto’s thyroiditis (HT) or the pancreas in type 1 diabetes mellitus (T1DM) or, on the other hand, affect multiple organs and tissues within the body, such as systemic lupus erythematosus (SLE) or rheumatoid arthritis (RA). Both types of ADs may exhibit a wide range of symptoms and can be challenging to diagnose and treat (4).

The exact causes of autoimmunity are not fully understood. Despite the presence of known genetic and epigenetic predisposing factors, environmental factors are believed to be an essential trigger of autoimmune response (5). The most well-studied example of such an external stimulus are pathogenic microorganisms or dysbiosis in commensal organisms, initiating either a non-specific immune response, or an immune response specific to self-antigens through molecular mimicry, like in the cases of the Epstein–Barr virus (6) or the group A streptococcus (7). Other well-known environmental factors leading to autoimmunity include smoking, diet, and drug administration (*e.g.*, immune checkpoint inhibitors therapy); and more such factors remain to be defined (3, 8).

ADs exhibit multi-phase dynamics and complex pathogenesis, including dysregulation in both adaptive and innate immune systems (9). At preclinical stages of the disease, autoantibodies – the hallmark of ADs – are expressed, in the absence of clinical signs and symptoms of autoimmune disorders such as fever, rash, and fatigue (10). A gradual involvement of functional elements of the immune system over time, such as aberrant B- and T-cells, leads to an eventual diversification of clinical phenotypes. However, despite the evolving understanding of the autoimmunity mechanisms, there are still numerous gaps in our knowledge regarding the role of specific immune components within a tangled network with multiple feedback loops in the pathophysiology of ADs, further complicating the management of disease progression (11).

The first-line therapy to control ADs include conventional immunosuppressive or anti-inflammatory therapies, such as corticosteroids and methotrexate, which dampen the overactivated immune system (12, 13). However, non-selective immunomodulators do not always provide sufficient benefit in a heterogeneous population of patients; also, their long-term administration leads to the appearance of side effects, the most common ones being an increased risk of infections and malignancies (14). Eventually, the accumulated knowledge on the pathophysiology of ADs has contributed to the development of targeted drugs with notably higher benefit-risk ratios. These selective immunotherapies are designed to suppress major pro-inflammatory signaling pathways by blocking inflammatory cytokines (*e.g.*, anti-interleukin (IL)-17 in psoriasis), target immune cells (*e.g.*, anti-B-cell activating factor (BAFF) in SLE), or intracellular kinases (*e.g.*, Janus kinase (JAK) inhibitors in RA). Despite the tremendous success of targeted therapies in ADs, unmet medical needs remain, in terms of long-term safety as well as overall efficacy, since many patients do not achieve disease remission (15). A deeper understanding of mechanisms and of their corresponding heterogeneity in the development of ADs would provide opportunities to overcome these limitations, ultimately leading to a more personalized and, therefore, a more efficient approach to treatment.

Mechanistic, physiologically-based mathematical modeling is widely accepted as a supportive tool for better understanding and interrogating diseases pathophysiology, given its ability to integrate biological and pharmacological knowledge with a quantitative description of the underlying processes, therefore making such modeling essential in enhancing decision-making at all stages of drug development (16, 17). For example, by reconstructing biochemical or signaling cascades *in silico*, a researcher may formulate and challenge theoretical hypotheses regarding the contribution of certain signaling components, to support the rational identification and validation of new drug targets (18, 19). Mechanistic modeling can also be employed to explore the impact of patient-specific factors, such as demographic characteristics and comorbidities, on drug action, potentially explaining the heterogeneity in treatment responses and providing options for adjustments or changes in treatment strategies. Modeling may further help in rationalizing the selection and application of drug combinations, in optimizing dosing schedule, and more (16). In summary, mechanistic mathematical modeling represents a tool to support the targeting of the right molecular pathway in a given disease context, with the right dose, and in the right patient population or phenotype (20).

The aim of this work was to conduct a systematic review and analysis of mathematical models of ADs focused on the mechanistic description of the immune system, to shape and consolidate the current landscape of quantitative knowledge on autoimmune processes, to outline most promising directions for further model developments, and to perform model-informed analyses aimed at supporting drug discovery and development against autoimmune pathologies.

## 2 Methods

A systematic literature search was performed in the PubMed database, to identify all mechanistic mathematical models with relevance to ADs. The search query consisted of two semantic components. The first one involved keywords related to autoimmunity, specifically, 184 disease-specific terms from the list of ADs (21) and general descriptors of autoimmune-related processes (*e.g.*, “autoimmune disease”, “autoinflammatory”, etc.). The second one was used to seek out studies on mechanistic, physiologically-based, or quantitative systems pharmacology (QSP) models. Additional search conditions were implemented, to eliminate records in non-English language and those focused on clinical trial results, reviews, or meta-analyses. To derive and compare the number of publications available in the PubMed database on mechanistic models *versus* empirical ones, another search was performed, with the second semantic component replaced with terms related to pharmacokinetics (PK) and pharmacodynamics (PD) analyses. The exact queries used are listed in the **Supplementary Methods**.

The search resulted in 500 potentially relevant publications (last accessed: 20 October 2023). Two authors (Y.U., A.V.) independently screened the articles for duplication and eligibility. Disagreements were resolved through discussion with independent reviewers (V.S., K.P.). A prerequisite for transferring an article into further analysis was the description of autoimmune-related processes at any level of generalization and organization (*e.g.*, molecular, cellular, tissue-level), including antigen presentation, cytokine production, immune complex (IC) formation, etc., using a mathematical framework such as ordinary differential equations (ODEs), partial differential equations (PDEs), and others. A model with any number of variables or parameters was considered mechanism-based as long as it included more than one of the above-mentioned components. In addition, articles featuring minor modifications vs. previously published models within a single AD were grouped and analyzed together.

Several classifications were used to categorize the models: by target organ or system, by indication, by mathematical method, and by associated data. The following criteria were applied to define the latter: if only clinical vs. preclinical data were used for calibration, fine-tuning of parameters, or model validation, the model was tagged to the respective group (*i.e.*, “Clinical” or “Preclinical”). If both types of data, preclinical and clinical, were used, or the parametrization was not within the scope of the research, “Combined” or “Not specified” tags were assigned.

Visualization and statistical analyses of the gathered data were carried out using reproducible scripts in the R Statistics software (version 4.0.2), using the R “tidyverse” package (version 1.3.0) (22). A network diagram of immune components was prepared using the R “igraph” package (version 1.2.7) (23).

## 3 Results

**Figure 1:**
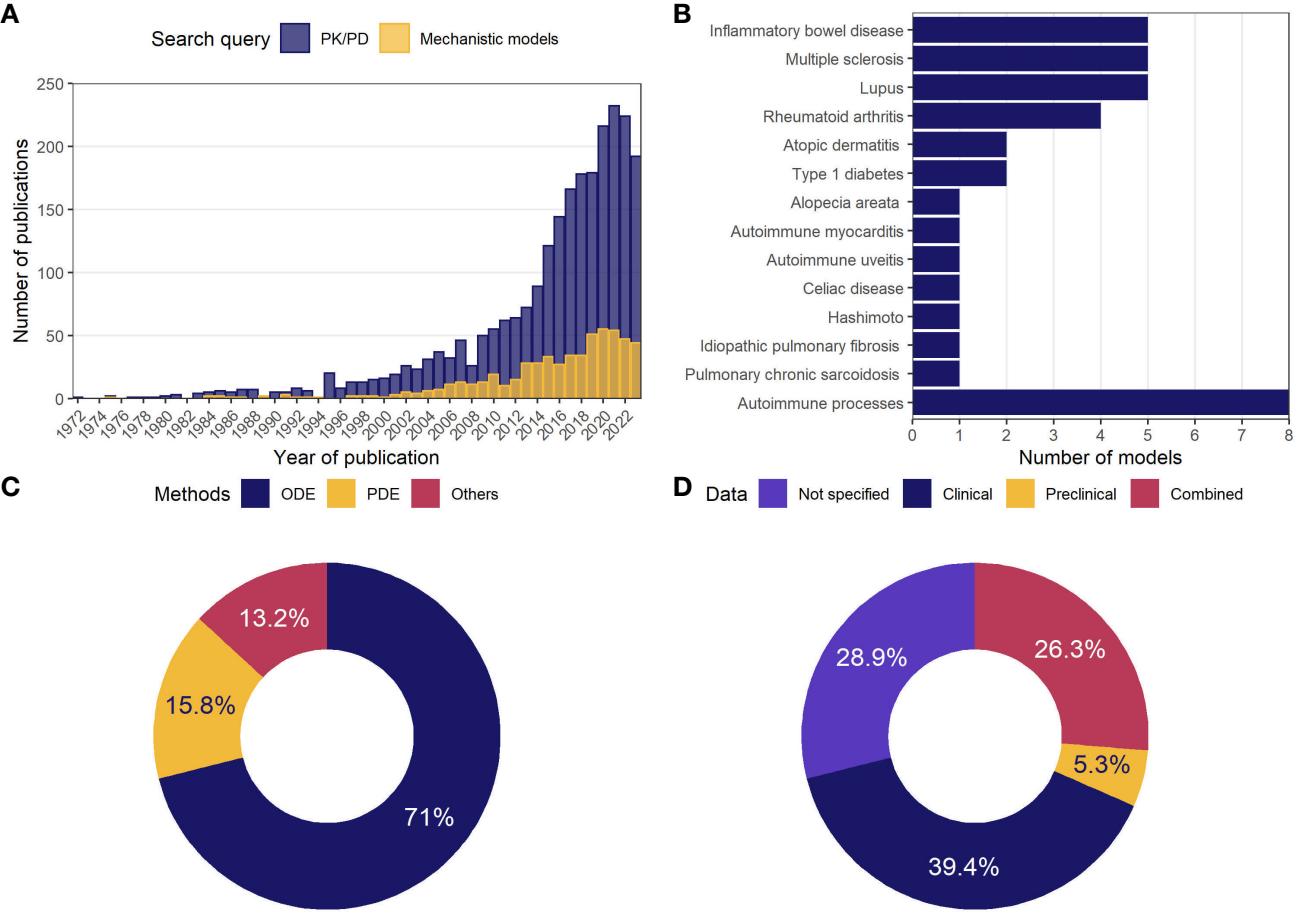
General statistics on model-based analyses in autoimmune diseases. **(A)** Number of publications in the PubMed database per year, following a search for mechanistic or PK/PD models of ADs; **(B)** number of mechanistic models identified through a systematic review and classified by indication; **(C)** percentage of mechanistic models using the described methodology; **(D)** percentage of mechanistic models by type of data used in model development or validation. AD, autoimmune disease; ODE, ordinary differential equations; PD, pharmacodynamics; PDE, partial differential equations; PK, pharmacokinetics.

To illustrate the growing body of publications related to mathematical modeling in ADs, the numbers of articles appearing in the PubMed database following the search queries for either PK/PD models or mechanistic models were visualized against years of publication (**Figure 1A**). According to the search results, the first papers matching the specifications date back to the 1970s. Since then, the number of relevant articles increased exponentially, reaching more than 200 publications per year by 2024. The ratio of studies appearing in the search for mechanistic models vs. empirical ones was small (~1-to-5).

Following a detailed investigation of these 500 publications potentially associated with mechanistic models, 47 studies were identified, which described the development, analysis, and application of 38 unique physiologically-based models. These 38 models were categorized according to 13 related AD indications, including alopecia areata, atopic dermatitis, autoimmune myocarditis, experimental autoimmune uveitis (EAU), celiac disease, HT, idiopathic pulmonary fibrosis (IPF), inflammatory bowel disease (IBD), multiple sclerosis (MS), pulmonary sarcoidosis, RA, SLE, T1DM, or classified into “Autoimmune processes” category if the model investigated autoimmunity in a more generic context rather than according to a specific AD (**Figure 1B**). The most frequently featured pathologies were IBD, MS, and lupus (5 models per disease), followed by RA (4 models), atopic dermatitis and T1DM (2 models each).

These models predominantly employed nonlinear systems of ODEs (71%) (**Figure 1C**). Some models were built using PDEs (15.8%); others featured integro-differential equations, Boolean networks, or Markov jump processes (13.2%). The models were developed primarily based on clinical data (39.4%); a small proportion of models made use of preclinical data only (5.3%), while 26.3% of model used a combination of clinical and preclinical data; 28.9% of models did not rely on quantitative data (**Figure 1D**).

**Figure 2:**
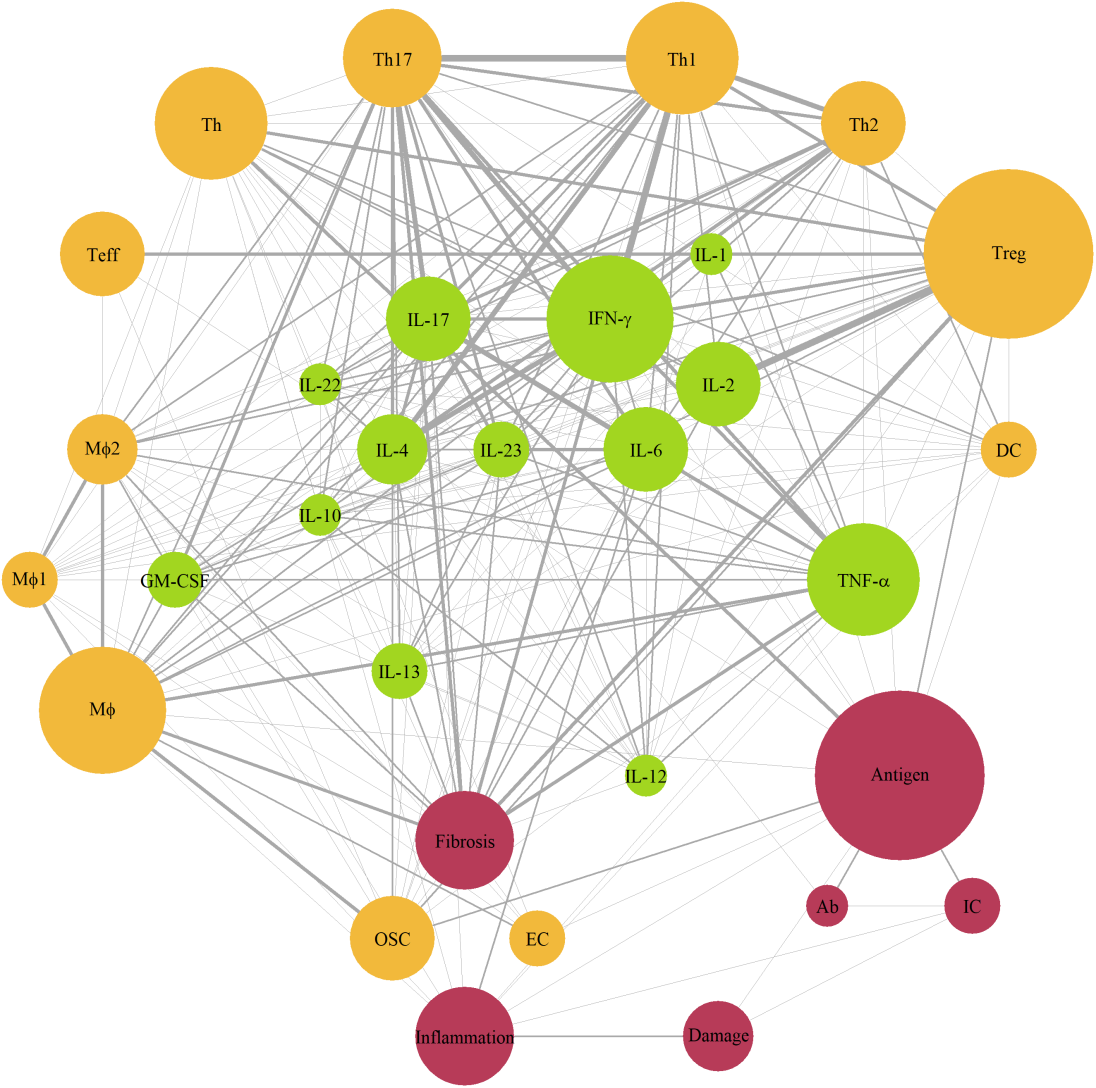
Network diagram of immune components represented in mechanistic models of autoimmune diseases. Green color – cytokines; yellow color – cells; red color – other components; size of a node – number of models with the component (3 to 11); edge width – number of interaction (3 to 6). Ab, antibody; APC, antigen-presenting cells, DC, dendritic cells; EC, epithelial cells; GM-CSF, granulocyte-macrophage colony-stimulating factor; IC, immune complex; IFN, interferon; IL, interleukin; Mφ, macrophages; OSC, organ-specific cells; Teff, effector T-cells; Th, T-helper cells; TNF-α, tumor necrosis factor-α; Treg, regulatory T-cells.

Given the multitude of ramifications found in the immune system, the mechanistic models we evaluated typically focused on specific aspects of autoimmune conditions and the related pathophysiology, and often at various levels of “mechanistic granularity”. To identify those components and interactions which occurred most frequently in the mathematical systems, we extracted all variables from the modeling papers – except for the Boolean network models – *i.e.*, 214 variables from 36 models; we then unified these variables into 60 terms and counted the incidence of terms occurring simultaneously within a single model (**Supplementary Material**; **Table 1**). If a term was encountered together with another term for at least 3 times, the corresponding interaction was added to the network diagram (**Figure 2**). The 31 nodes which appeared on the diagram were next divided into 3 categories, by biological origin (*i.e.*, cellular, or molecular) or effect (*e.g.*, fibrosis, inflammation, tissue damage). The most frequently occurring terms were antigen and regulatory T-cells (Treg) (12 cases), followed by interferon (IFN)-γ and macrophages (Mφ) (9 cases each), tumor necrosis factor-α (TNF-α), T-helper cells (Th) and Th1, (8 cases each), and Th17, inflammation, fibrosis (7 cases each). Consequently, most frequent interactions (at least 5 per edge) were observed between T-cells (Tregs, Th1, Th17) and related cytokines (IFN-γ, IL-2, IL-4, IL-6, IL-17).

**Table 1:** Summary of mechanistic models of autoimmune diseases.

Reference	Indication	Description	Application	Key limitations
Systemic autoimmune diseases				
Lupus				
Ruiz-Cerdá et al. (24)	Systemic lupus erythematosus	A Boolean network model of antigen presentation	Identification of promising biological targets (including their combinations) as well as conditions defining the treatment response.	1. Qualitative nature of the Boolean network models. 2. The scope of the system is limited to the antigen presentation.
Yazdani et al. (25)	Systemic lupus erythematosus	Mechanistic ODE-based model of SLE at different stages of the disease progression	Model-based assessment of immune system condition at different stages of SLE (*e.g.*, tolerance breach, occurrence of fares, etc.) and the effect of mesenchymal stem cell therapy.	1. Qualitative rather than quantitative approach to model parametrization. 2. Utilization of generic variables not directly associated with laboratory measurements.
Budu-Grajdeanu et al. (26)	Lupus nephritis	Generic 4-ODE system describing the dynamics of ICs, anti- and pro-inflammatory mediators, and tissue damage	Exploration of intersubject variability associated with the treatment benefit.	1. Utilization of generic variables not directly associated with laboratory measurements. 2. The model was calibrated using the data on 4 subjects.
Hao et al. (27) Karagiannis et al. (28)	Lupus nephritis	A system of convection-diffusion equations describing the progression of renal fibrosis	Identification of promising biological targets for the treatment of renal fibrosis.	Limited application of clinical data for model development.
Gao et al. (29)	Systemic lupus erythematosus	The effect of exogenous IL-2 on the ratio between conventional T-cells and Tregs described using ODE framework	1. Defining therapeutic window for IL-2 in patients with SLE. 2. Search for covariates and predictive biomarkers associated with the IL-2 treatment.	The scope of the model is focused on the clinical issues specific to the IL-2 therapy.
Rheumatoid arthritis				
Rullmann et al. (30)	Rheumatoid arthritis	Application of the Entelos^®^ RA PhysioLab^®^ platform	1. Validation of potential biological targets. 2. Prediction of the effect of hypothetical anti-IL-15 and anti-IL-12 treatment on synovial cell density and cartilage degradation rate.	Source code is not available.
Moise et al. (31)	Rheumatoid arthritis	A system of PDEs describing rheumatic joint (cartilage, synovial membrane, synovial fluid compartments) in the chronic state of RA	1. Mathematical evaluation of the disease state. 2. Evaluation of the effect of conventional therapies (methotrexate, infliximab, tocilizumab), and hypothetical ones (anti-IL-23 and anti-IL-17), including their combinations.	1. Simplified geometry of the joint. 2. Focus on the behavior of the mathematical system without direct association with the clinical data (*e.g.*, model validation or parameter calibration against the observed data).
Nakada et al. (32)	Rheumatoid arthritis	Five target engagement models of cytokines and respective antagonists in ODEs, linked with the CRP dynamics	A robust quantitative model calibrated and validated using data from multiple clinical trials and applied to explain interpatient variability in support of dosing strategy optimization for the considered compounds.	Limited roster of biological entities (*e.g.*, immune cells not included) and associated therapies.
Meyer-Hermann et al. (33)	Rheumatoid arthritis	A system of ODEs quantifying circadian variations of cortisol, noradrenaline, and TNF-α in healthy subjects and patients with RA	1. Evaluation of the cortisol and noradrenaline response under anti-TNF-α treatment. 2. Optimization of the clock time of drug administration.	Immune response is represented only by TNF-α.
Organ-specific autoimmune diseases				
Gastrointestinal tract				
Wendelsdorf et al. (34)	Inflammatory bowel disease	An ODE model that describes innate and adaptive immune response to bacteria stimuli in IBD across gut lumen, lamina propria, and mesenteric lymph node	1. Testing different rescue strategies (*e.g.*, targeting Mφ or Treg) for IBD *in silico*. 2. Explaining the mechanisms behind PPARγ-mediated IBD prevention.	1. Relatively high-level description (*i.e.*, using generic variables) of the biological mechanisms. 2. The model was developed and validated using preclinical data.
Lo et al. (35, 36) Park et al. (37)	Inflammatory bowel disease	An ODE model of T-cell polarization in IBD	1. Exploration of the cytokine and transcription factor balance associated with proper and pathological T-cell immune response. 2. Evaluation of the treatment effect of anti-cytokine therapies across 4 sub-groups of IBD patients defined by the ratio of Th1 and Th2 transcription factors.	1. The quantities of T-cells are represented by transcription factors. 2. Part of the model parameters were derived from the models of other diseases.
Dwivedi et al. (38)	Inflammatory bowel disease	Mechanistic model of IL-6 signaling adapted for IBD	Benchmarking different targets for IL-6 signaling disruption based on CRP concentrations as a marker of inflammation.	The focus of the modeling is only on the IL-6 pathway.
Rogers et al. (39, 40)	Inflammatory bowel disease	Comprehensive mechanistic model that includes detailed description of innate and adaptive immune response in IBD developed in ODEs	1. Explaining the mechanisms behind IL-17 inhibition-mediated disease worsening. 2. Utilizing virtual population approach to predict the number of responders to different treatment options, including the combination of anti-TNF-α and anti-IL-12p40 compounds.	Large scale of the model (116 reactions and 334 parameters) which makes it hard to adapt and qualify.
Balbas-Martinez et al. (41, 42)	Inflammatory bowel disease	Boolean network model with 43 nodes spread across lymph node, blood, lymph circulatory system, and gut lumen compartments, subsequently expanded with an ODE sub-module	1. General description of the IBD condition and the effect of several therapeutic interventions using Boolean network approach. 2. Predicting the effect of recombinant human IL-10 administration on other cytokines in the system.	Qualitative nature of the Boolean network models.
Demin et al. (43)	Celiac disease	A mechanistic model in ODEs attempting to describe immune response in lamina propria provoked by the consumption and deamidation of gluten	Benchmarking potential biological targets, including transglutaminase-2 inhibitors, for the treatment of celiac disease.	Limited clinical data available for model validation.
Nervous system and eyes				
Nicholson et al. (44)	Autoimmune uveitis	A 10-ODE model that describes the processes of antigen presentation and T-cell activation on both sides of the blood-retina barrier	Evaluation of the impact of blood-retina barrier permeability and other factors (*i.e.*, APC production) on the disease state.	Biological mechanisms represented in the model are simplified and limited to several generic variables.
Moise et al. (45)	Multiple Sclerosis	A 27-PDE model describing immune and inflammatory interactions within the focal plaque	Quantifying the impact of stand-alone treatment and combinations of IFN-β, glatiramer acetate, natalizumab, and dimethyl fumarate on plaque growth	1. Simplified geometry of the plaque. 2. Limited application of clinical data for model development.
Vélez de Mendizábal et al. (46)	Multiple Sclerosis	A generic model of the cross-regulation between regulatory and effector T-cell in MS in ODEs	Exploring the mechanisms behind different MS phenotypes.	1. Limited number of immune response components considered in the model. 2. Focus on the behavior of the mathematical system with limited application of the clinical data.
Kannan et al. (47)	Multiple Sclerosis	A generic 4-ODE model of inflammatory and anti-inflammatory components, demyelination, and neuronal death	Exploring the mechanisms behind different MS phenotypes.	1. Focus on the behavior of the mathematical system with limited application of the clinical data. 2. Utilization of generic variables not directly associated with laboratory measurements.
Gross et al. (48)	Multiple Sclerosis	A probabilistic model describing transmigration and differentiation of lymphocytes in the CNS	Assessing the effectiveness of immune-modulating therapies without the need for lumbar punctures.	Limited clinical data available for model validation.
Broome et al. (49)	Multiple Sclerosis	An ODE systems model describing the interactions between reactive oxygen and nitrogen species, the permeability transition pore, apoptotic factors, and eventual cell death in oligodendrocytes	Identification of the promising biological targets for the treatment of MS.	Limited number of immune response components considered in the model due to the biochemical scope of the model.
Skin				
Dobreva et al. (50, 51)	Alopecia areata	An ODE model describing hair follicles dystrophy as a function of several immune components, including autoreactive T-cells, IFN-γ, MHC-I and immune privilege guardians	Exploring hypotheses on disease pathogenesis *via* mathematical description of the alopecia areata progression.	1. The model was developed using preclinical data. 2. Focus on the behavior of the mathematical system with limited application of the clinical data. 3. Limited number of immune response components considered in the model.
Tanaka et al. (52) Domínguez-Hüttinger et al. (53) Christodoulides et al. (54)	Atopic dermatitis	An ODE-based system describing the four phenotypes typical for atopic dermatitis patients as a function of pathogen load, immune response, and the strength of skin barrier	Optimization of treatment schedule and combinatory effect of antibiotics, emollients, and corticosteroids in terms of transition between pathogenic and healthy phenotypes.	1. No clinical data was used in model development. 2. Lack of temporal components in drug PK and several major factors affecting disease phenotype.
Miyano et al. (55)	Atopic dermatitis	Modeling of the EASI efficacy score as a function of pathogen load, cytokine concentration, and skin barrier integrity using ODE framework	1. Indirect comparison of efficacy across 9 anti-cytokine drugs. 2. Optimization of therapeutic strategy for dupilimab-resistant patients and search for predictive biomarkers associated with the dupilumab treatment-mediated response.	1. Covariance in model parameters was not considered when generating virtual patient population. 2. Each cytokine independently affects skin barrier integrity and infiltration by pathogens. 3. PK/PD relationship was not explored was only maximum dose of investigated compounds was considered.
Endocrine system				
Magombedze et al. (56)	Type 1 diabetes mellitus	A 6-ODE model capturing changes over time in β-cells, effector T-cells, Tregs, Mφ (resting and activated), and autoantigen	Exploring the mixture of factors causing the onset and progression of T1DM, particularly the impact of Tregs on the suppression of autolytic T-cells.	1. Limited number of immune response components considered in the model. 2. Focus on the behavior of the mathematical system with limited application of the clinical data.
Jaberi-Douraki et al. (57)	Type 1 diabetes mellitus	A concise model of integro-differential equations reflecting the interplay between β-cell, effector T-cells and Tregs along with antigen and IL-2	Describing the onset and progression of T1DM and categorizing individuals based on the balance between effector T-cells and Tregs, and T-cells avidity.	1. Limited number of immune response components considered in the model. 2. Focus on the behavior of the mathematical system with limited application of the clinical data.
Salazar-Viedma et al. (58)	Hashimoto’s thyroiditis	A 4-ODE model representing Th1, Th17, thyrocytes and gut microbiome dynamics	Identifying the scenarios in Th and intestinal microbiota imbalance causing the development and progression of HT.	1. Limited number of immune response components considered in the model. 2. Focus on the behavior of the mathematical system with limited application of the clinical data.
Lungs				
Aguda et al. (59) Hao et al. (60)	Pulmonary chronic sarcoidosis	A system of convection-diffusion equations describing cells dispersion and cytokine diffusion within granuloma	Benchmarking the effect of cytokine-targeting drugs (anti-TNF-α, anti-IL-12, anti-IFN-γ, TGF-β enhancement) on the size of sarcoid granulomas.	1. Limited application of clinical data for model development. 2. Simplistic geometrical representation of the granuloma as a sphere with uniform distribution of Mφ, Th1, Th17 and Treg.
Hao et al. (61)	Idiopathic pulmonary fibrosis	A system of convection-diffusion equations describing the progression of pulmonary fibrosis	Benchmarking the effect of cytokine-targeting drugs (anti-TNF-α, anti-PDGF, anti-IL-13, anti-TGF-β) on pulmonary fibrosis progression.	1. Limited application of clinical data for model development. 2. Simplistic geometrical representation of the lung tissue.
Cardiovascular				
van der Vegt et al. (62)	Autoimmune myocarditis	A 4-ODE model capturing the development of autoimmune myocarditis under treatment with immune checkpoints inhibitors	Establishing individual patient characteristics reflected in the values of several key model parameters leading to the development of autoimmune myocarditis under treatment with nivolumab or ipilimumab.	1. Biological mechanisms represented in the model are simplified and limited to several generic variables. 2. Focus on the behavior of the mathematical system with limited application of the clinical data.
General autoimmune reactions				
Head et al. (63)	Autoimmune processes	A systems model describing IC formation, opsonization and clearance	Evaluation of mathematical conditions resulting in an increased production ICs to identify the states associated with the high risk of the development of IC-mediated autoimmune disease.	Focus on the behavior of the mathematical system with limited application of the clinical data.
Arazi et al. (64)	Autoimmune processes	A generic 3-ODE model reflecting the quantities of autoreactive B-cells, autoantigen, and ICs	Establishing how initial conditions affect the degree of IC-mediated inflammation and which processes contribute the most to it.	1. Focus on the behavior of the mathematical system with limited application of the clinical data. 2. Limited number of immune response components considered in the model.
Iwami et al. (65)	Autoimmune processes	A 3-ODE model of immune cells, target cells, and damaged cells	Exploring different functional relationships and parameter spaces explaining different states of immune tolerance/dormancy and repeated flare-ups in AD.	1. Focus on the behavior of the mathematical system with limited application of the clinical data. 2. Utilization of generic variables not directly associated with laboratory measurements.
Khailaie et al. (66)	Autoimmune processes	A mechanistic model of the interplay between conventional and Treg	Delineating three regimes of immune activation (complete lack of response, the first peak following by complete clearance of the antigen and chronic persistence of the antigen) primarily through the differences in the renewal rate of naïve T-cells and resting Tregs and antigen-stimulation thresholds.	1. Focus on the behavior of the mathematical system with limited application of the clinical data. 2. Limited number of immune response components considered in the model.
Louzoun et al. (67)	Autoimmune processes	A model representing the dynamics of Th1, Th2, Mφ their cytokines, as well as naïve T-cells and pooled Teff and Treg	Characterizing the progression of Th1-type autoimmune diseases from Th1 to Th2 steady-state	1. Focus on the behavior of the mathematical system with limited application of the clinical data. 2. Utilization of generic variables not directly associated with laboratory measurements.
Hara et al. (68)	Autoimmune processes	A 4-ODE model describing the interplay between Th (reactive to self-antigen or viral infection), virus, and memory T cells	Identifying the conditions that trigger autoimmunity in response to viral infection	1. Focus on the behavior of the mathematical system with limited application of the clinical data. 2. Utilization of generic variables not directly associated with laboratory measurements.
Ramos et al. (69)	Autoimmune processes	A system of integro-differential equations and ODE describing the interaction of self-APC, self-reactive T cells and immunosuppressive cells	Exploring of mathematical conditions resulting in the regulatory effect of immunosuppressive cells on the proliferation and activation of self-reactive T cells in an autoimmune reaction.	1. Focus on the behavior of the mathematical system with limited application of the clinical data. 2. Limited number of immune response components considered in the model.
Valeyev et al. (70)	Autoimmune processes	An ODE model representing two exemplar immune cell populations and their mutual regulation through cytokine signalling	Defining mathematical conditions leading to the oscillating or trigger-based pathological disease phenotype.	1. Focus on the behavior of the mathematical system with limited application of the clinical data. 2. Utilization of generic variables not directly associated with laboratory measurements.

CNS, central nervous system; CRP, C-reactive protein; HT, Hashimoto’s thyroiditis; IBD, inflammatory bowel disease; IC, immune complex; IFN, interferon; IL, interleukin; MHC, major histocompatibility complex; MS, multiple sclerosis; Mφ, macrophages; ODE, ordinary differential equation; PDE, partial differential equation; PK, pharmacokinetics; RA, rheumatoid arthritis; SLE, systemic lupus erythematosus.; T1DM, type 1 diabetes mellitus; Th, T-helper cells; TNF-α, tumor necrosis factor-α; Treg, regulatory T-cells.

**Figure 3:**
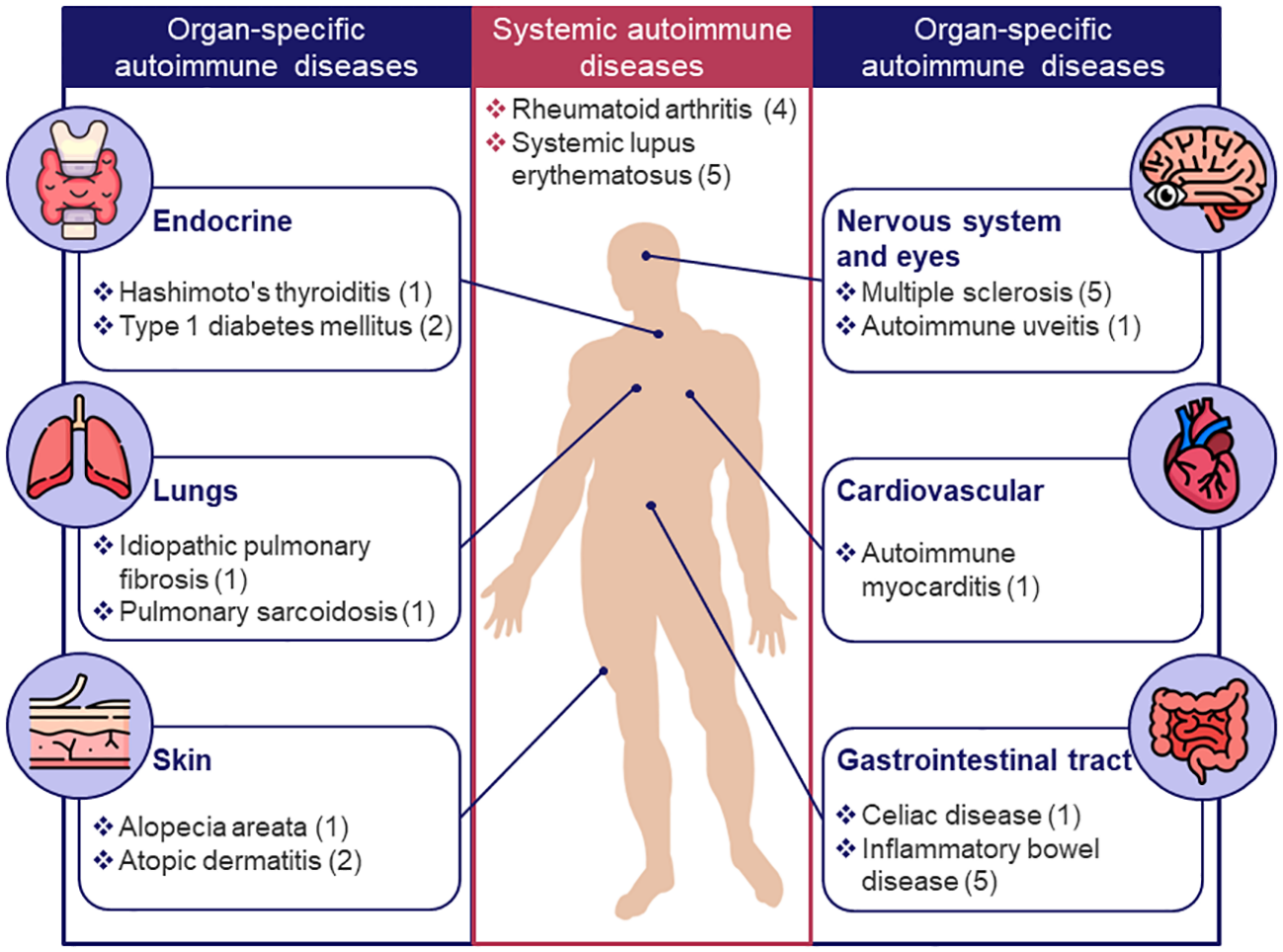
Classification of existing models for autoimmune diseases by target organ or system. Only diseases for which mechanistic models were identified are shown; the number of models for a given disease is indicated in brackets.

Next, the 30 models associated with specific diseases (see **Figure 1B**) were categorized into 2 groups: systemic- and organ-specific. The latter category was, in turn, separated into 6 subgroups: endocrine, lungs, skin, nervous system and eyes, cardiovascular, and gastrointestinal (GI) tract (**Figure 3**). Mathematical systems describing autoimmune processes in a more generic sense (8 models) were kept in a separate category. All 38 models were subjected to a comprehensive evaluation encompassing their description, applications, and limitations, summarized in **Table 1** and in the text below.

### 3.1 Lupus

Lupus is a chronic systemic AD with heterogeneous clinical manifestations, ranging from mild joint and skin abnormalities to life-threatening kidney, cardiac, or central nervous system (CNS) impairment (71). The etiology and pathophysiology of this AD is complex and is still poorly understood. Multiple genetic, immunological, and environmental factors may influence the loss of immunological tolerance against self-antigens, leading to the activation of autoreactive T- and B-cells. Dysregulation in T-cell response results in an imbalance in cytokine production, attracting an increasing number of immune cells to the affected tissue and provoking further inflammation. Moreover, pathological B-cells produce autoantibodies that cause organ damage by IC deposition and complement system activation (72, 73).

The first endeavor in providing a comprehensive depiction of systemic inflammation in SLE was a Boolean network model developed by Ruiz-Cerdá and colleagues (24). This model employed 52 nodes, to represent components of antigen presentation by antigen-presenting cells (APC) to T-cells, with 254 interactions describing activation, inhibition, upregulation, or downregulation processes between nodes. By knocking out or overstimulating isolated nodes, the authors identified and classified perturbations leading to a “lupus-like” phenotype in a virtual subject.

A recent example of another self-contained SLE model, built upon ODEs, is the work by Yazdani et al. (25). These authors created a system that delineates various stages of SLE progression, encompassing the entire immune response in 13 variables: 8 of these are generic (*e.g.*, “proinflammatory mediators”, “damaged tissue”), and the other 5 reflect specific immune response components: autoantibodies, antigens, IC, and mesenchymal stem cells, as a potential approach for SLE treatment. Model parameters were manually tuned to reproduce key immunological patterns at different phases of SLE, such as tolerance breach, the onset of systemic inflammation, the development of clinical signs, and the occurrence of flares and remissions.

Subsequently, this model underwent modifications through the incorporation of a tissue inflammation submodule, which, in turn, was adopted from another model developed by Budu-Grajdeanu and colleagues for lupus nephritis (LN) (26). This model describes changes over time for 4 variables: IC, anti-inflammatory mediators, pro-inflammatory mediators, and damaged tissue. The first two variables are generic (*i.e.*, not associated with specific biological entities), whereas the last two variables represent urine biomarkers measured in routine clinical practice: urine monocyte chemotactic protein-1 (uMCP-1) and urine protein-to-urine creatinine ratio, respectively. 4 sets of individual parameters were derived from the model calibration procedure, based on individual data from 4 subjects with SLE – a first step towards the quantification of inter-individual variability.

The pathophysiology of LN, specifically in relation to the progression of renal fibrosis, is also the focus of another series of articles published by Hao and colleagues (27, 28). The publications describe the system as a set of 11 PDEs depicting the dispersion of various types of cells involved in fibrosis, across rectangular cross-section of the renal cortex, and regulated by a network of cytokines and growth factors, including uMCP-1, platelet-derived growth factor (PDGF), transforming growth factor-beta (TGF-β), matrix metalloproteinase (MMP), and tissue inhibitor of metalloproteinases (TIMP). Through a sensitivity analysis, two parameters were identified as most influential for the formation of interstitial fibrosis and were estimated based on uMCP-1 time profiles measured in 84 subjects. Between-subject variability was taken into account by dividing the subjects according to their severity of fibrosis (low, intermediate, or high). The estimated two parameters affect the production of TGF-β by tubular epithelial cells (EC) and uMCP-1 synthesis by Mφ; modulating these has been shown to provide a potentially beneficial therapeutic strategy for mitigating renal fibrosis.

As opposed to a rather qualitative assessment of drug effects in the aforementioned models, another model considered in this section was specifically designed to quantify the treatment effect of exogenous IL-2 therapy in patients with SLE (29). The model by Gao et al. consists of 10 ODEs and focuses on a limited number of immune components directly associated with the therapeutic effect of IL-2, including conventional T-cells (Tcon), Treg, and natural killer (NK) cells – also taking into account differences in IL-2 receptor densities on each cell type and the formation of receptor-ligand complexes. The model was applied to select an effective range of IL-2 concentrations for treating SLE patients based on their Tcon/Treg ratio. Furthermore, factors affecting the exposure-response relationship were identified, and a new prognostic biomarker (Treg/CD4+ T-cells ratio) was proposed to separate responders and non-responders prior to treatment initiation.

### 3.2 Rheumatoid arthritis

RA is a chronic, systemic AD characterized by joint inflammation, synovial hyperplasia, and the progressive destruction of cartilage and bone, leading to disability and impaired quality of life. The disease pathophysiology is complex and involves a wide array of elements of the innate and adaptive immune responses, including Mφ, dendritic cells (DC), NK cells, T- and B-cells, fibroblast-like synoviocytes, as well as cytokines such as TNF-α and IL-6, along with others: IL-7, IL-15, IL-17, IL-21, IL-23, and granulocyte-macrophage colony-stimulating factor (GM-CSF) (74–77). Similarly to other ADs, the heterogeneity in RA pathophysiology represents a major confounding factor for the development of targeted RA therapies. Despite several drugs available on the market, including anti-TNF-α (infliximab, etanercept), anti-IL-6 (tocilizumab), anti-JAK (tofacitinib), and anti-CD20 (rituximab), their effectiveness is limited to subgroups of patients, while their administration is associated with serious side effects such as ulcers, fatigue, reduced immunity to infections, and osteoporosis (78, 79).

In our search, we identified 4 diverse examples of mechanistic models applied to study RA disease and associated treatments. The first study by Rullmann, published in 2005, describes the application of the Entelos^®^ (80) RA PhysioLab^®^ platform to validate potential biological targets as well as to predict the effect of hypothetical anti-IL-15 and anti-IL-12 treatments on synovial cell density and cartilage degradation rate (30). The model consists of ODEs, is claimed to include several dozens of soluble factors, cell surface molecules, and numerous types of cells in rheumatoid joint, and can simulate virtual patients with various properties; the lack of the source code, however, prevents a further detailed evaluation.

In 2018, Moise and Friedman developed their own model of a rheumatic joint in a chronic RA state, using PDEs (31). The system describes the dynamics, distribution, and cross-influence of Th17 cells, fibroblasts, Mφ, and associated cytokines and chemokines (*e.g.*, IL-17, GM-CSF, IL-6, fibroblast growth factors, TNF-α, etc.) across cartilage, synovial membrane, and synovial fluid compartments. In addition to an evaluation of model behavior over time without treatment (*i.e.*, disease progression), the model was used to test theoretical effects of conventional therapies (methotrexate, infliximab, tocilizumab), and hypothetical ones (anti-IL-23 and anti-IL-17), including their combinations. Cartilage degradation was used as a surrogate measure of disease status.

One of the more recent works published by Nakada and Mager (2022) describes a mechanistic ODE model focused on the interplay between several key cytokines associated with RA pathophysiology: IL-6, IL-17, TNF-α and IL-1 – complemented with the PK of therapeutic agents such as tocilizumab, secukinumab, infliximab, canakinumab, and anakinra (32). The dynamics of each cytokine and respective antagonist(s) were described by a target-mediated drug disposition module. Modules were connected through a network of feedback mechanisms, with, at the top, C-reactive protein (CRP) turnover as a marker of inflammation. The model is highly quantitative, being informed by data from multiple clinical trials based on the above-mentioned compounds and validated using external datasets not used for model calibration. From a drug development perspective, the model was applied to identify covariates (primarily baseline cytokine concentrations) explaining inter-patient variability in anti-inflammatory effects of the considered anti-cytokine therapies.

The fourth and final model in this section does not focus on inflammatory components but rather on circadian variations of cortisol, noradrenaline, and TNF-α as key players, respectively, of the endocrine, nervous, and immune system, in healthy subjects and patients with RA (33). As such, the characterization of daily oscillations of these markers under anti-TNF-α treatment can be considered as the main output of this model-based research. In addition, the authors demonstrated, *via* simulations, that glucocorticoid treatment between midnight and 2:00 AM would result in the strongest inhibitory effect on TNF-α secretion.

### 3.3 Gastrointestinal tract

The GI tract is a chain of interconnected organs that consists of the oral cavity, pharynx, esophagus, stomach, small intestine, large intestine, and anal canal. It is a system constantly exposed to the elements of the environment, simultaneously providing an organism with nutrition while keeping numerous pathogens at bay. Multiple ADs (*e.g.*, autoimmune hepatitis, autoimmune pancreatitis, SLE) exhibit symptoms associated with the GI tract (81), while others are directly caused by abnormal immune reactions within the organ system. Most common examples of the latter are IBD, which includes Crohn’s disease (CD) and ulcerative colitis (UC), and celiac disease (82). A large volume of empirical knowledge has accumulated over the years, allowing us to define the pathogenesis of these conditions with relative certainty. Firstly, the right balance between a proper immune response to disease agents and the lack of such for food-related antigens can be challenged by an increased permeability in the gut and a loss of immune tolerance to self-antigens (83). Secondly, inflammation may be provoked by the dysregulation of Th processes, *e.g.*, an increased activation of Th1/Th17 or deactivation of the Th2/Treg pathway (84).

One of the first mechanistic models of IBD by Wendelsdorf and colleagues attempts to quantify these processes across three physiological compartments: the lumen, the lamina propria, and the mesenteric lymph node (34). The model describes the amplification of innate and adaptive immune responses in response to bacteria stimuli which may cause depletion of the epithelial lining. The model operates with 30 variables which can be roughly divided between Mφ (M0, M1, M2), DC, and T-cells (both pro- and anti-inflammatory), as well as cytokines (activating and deactivating). The model allows for a comprehensive sensitivity analysis and a high-level hypothesis evaluation related to general interactions between immune cells. For example, the model was applied to propose rescue strategies that remove M1 from the site of infection, which explains the mechanisms of proliferator-activated receptor-γ-mediated IBD prevention; the model also describes effects of chemokine and cytokine deprivation that allow for Mφ to remain activated. However, the price for the large scale of the model is a rather abstract description of mechanisms, a limitation which has been partially compensated for by subsequent modeling research.

For example, Lo and colleagues focused on aspects of T-cell polarization during the initial steps of the inflammatory processes in IBD, by modeling Th1, Th2 and Treg population densities as a function of concentrations of transcription factors (T-bet, Gata3 and Foxp3) and four cytokines (IFN-γ, IL-4, TGF-β and IL-2) (35). The system was explored primarily along two scenarios: bacterial infection and protozoan infection - under normal and abnormal immune responses, through the modulation of model parameters. It provides a better understanding of the delicate balance between cytokine and transcription factors required for proper T-cell immune response in the gut and the consequences of its disruption. At the same time, the model lacks several essential mechanistic components, including the polarizing effect of cytokines produced by activated Mφ. This limitation was addressed in subsequent work by the same authors, where they expanded on the developed model and added several additional immune components, including Th17, M1 and M2 Mφ, along with IL-6, IL-10, IL-12, IL-21 and TNF-α (36). The model was applied to characterize 4 subgroups of patients with CD, as defined by the ratio of Th1 and Th2 transcription factors, relative to values in healthy volunteers, based on biopsy data. A hypothetical anti-TNF-α therapy was then tested in each subgroup, to find the population most sensitive to the treatment. The article by Park et al. capitalizes on the work by Lo and colleagues and explores the effect of anti-IL-12 and pro-IL-10 treatment along with TNF-α suppression in the same system, with 4 cohorts of patients differing in their quantities of Th1 and Th2 (37).

The model by Dwivedi et al. is another example of a systems model focused on cytokine effects, IL-6 in particular (38), which contribute to Th17 differentiation (85). This network of ODEs is based on a previously developed model of IL-6-mediated immune signaling, includes 3 compartments (liver, GI tract and circulation), and describes IL-6 binding with its IL-6 receptor (IL-6R) and gp130. The model was validated based on tocilizumab (an anti-IL-6R antibody) data and used to benchmark different targets for IL-6 signaling disruption using circulating levels of CRP, a well-established marker of inflammation, as the primary pharmacodynamic measurement.

The most ambitious attempt to quantify and integrate all pathways and processes described above and beyond can be attributed to a series of papers by Rogers and colleagues, who describe a model with 116 reactions and 334 parameters (39, 40). This model includes multiple cell types associated with innate (DC, Mφ, NK cells, and neutrophils) and adaptive immunity (Th1, Th2, Th17, and Tregs), more than 10 cytokines, CRP and fecal calprotectin as dependent variables, and was parameterized for three types of subjects (healthy, CD, UC), using a technique proposed by Allen et al. (86) to generate virtual populations with baseline biomarker levels corresponding to actual observations. Moreover, the model includes PK models of anti-TNF-α (infliximab), anti-IL-12p40 (ustekinumab), anti-IL-23 (risankizumab and brazikumab), and anti-IL-6 (PF-04236921) compounds. In the first part of their work, the authors performed a sensitivity analysis to identify key mechanisms affecting fecal calprotectin and CRP; they then applied the model to understand mechanisms underlying the worsening of CD, in the case of IL-17 inhibition. In the second part, the model was thoroughly validated against data on multiple existing compounds and was used to predict responder rates based on biomarker cutoffs as well as the effect of combined anti-TNF-α and anti-IL-12p40 treatments.

Another example of a comprehensive, yet qualitative model of IBD is based on a Boolean network, rather than ODEs, created by Balbas-Martines et al. (41). It consists of 43 nodes spread across lymph node, blood, lymph circulatory system, and gut lumen, with MMP being the main marker of tissue damage in the system. The model was shown to correctly reflect the lack of response in MMPs under IL-10 overexpression, IL-17 or IFN-γ knockout and, in turn, demonstrates marked improvement in disease condition mediated by TNF-α suppression or granulocyte and monocyte apheresis. Subsequently the system was ameliorated to a hybrid model of ODEs and Boolean processes, to allow for the characterization of the magnitude and dynamics of IL response, to predict the effect of recombinant human IL-10 administration on 14 cytokines involved in CD pathogenesis (42).

Aside from IBD, which holds the central place as the subject of multiple model-based research, other GI tract-related ADs, such as celiac disease, have been represented by a single model (43). It is similar in principle to the model by Wendelsdorf et al. (34) and attempts to describe the dynamics of several key immune components and disease progression elements, including gluten deamidation, antigen presentation, T-cell activation, and the formation of auto-antibodies, in the lamina propria. The model was applied to evaluate potential targets for the treatment of celiac disease, among which gluten-peptide analogues showed the strongest effect on antibodies and the villous area, although the clinical efficacy of DQ2 or DQ8 gene analogues remains, to date, unknown (87).

### 3.4 Nervous system and eyes

Various ADs, including RA, SLE, and MS, might have devastating effects on vision (88–90). However, the eyes themselves can be the target of an autoimmune condition such as, for example, EAU, which has been explored via the means of mathematical modeling by Nicholson et al. (44). Similarly to many other organ-specific autoimmune disorders, EAU is caused by an abnormal influx of effector cells into the target organ. A distinctive feature of EAU, however, is the presence of the blood-retina barrier. Thus, Nicholson et al. developed a minimalistic model of 10 ODEs that describes essential processes of antigen presentation and T-cell activation on both sides of the blood-retina barrier, with corresponding transitions. This allowed the authors to evaluate the impact of permeability on disease state and highlight other factors (*i.e.*, APC production) which may contribute significantly to abnormal inflammation.

The nervous system, comprised of the CNS and the peripheral nervous system, regulates the functionality of organs through the processing of internal and external information conveyed via electrical impulses along an extensive network of nerve endings. MS, a prevalent autoimmune neurological disorder, manifests itself as demyelinating lesions in the CNS (91, 92). From a clinical perspective, MS displays notable heterogeneity, divided among a primarily progressive multiple sclerosis (PPMS) type, which is characterized by a gradual accumulation of clinical disabilities, and a relapsing-remitting multiple sclerosis (RRMS) type, which is characterized by disease exacerbations followed by periods of remission – although often switching to a secondary progressive multiple sclerosis (SPMS) type in the long-term (93, 94). The immune pathophysiology of MS involves bidirectional interactions among peripheral immune cells (T-cells, B-cells, myeloid cells) and resident CNS cells (microglia, astrocytes). For the RRMS type, focal inflammatory demyelination caused by peripheral immune cells infiltrating the CNS is believed to be the cause of relapses, whereas for the progressive type, diffuse tissue damage of white and gray matter is predominant (95).

In their model, Moise and Friedman focused on delineating immune and inflammatory interactions within the focal plaque, quantifying their impact on plaque growth (45). The authors proposed a model based on 23 PDEs encompassing the dynamics of Th, cytotoxic T-cells, Mφ, astrocytes, oligodendrocytes, chemokines, and 8 pro- and anti-inflammatory cytokines. Additionally, 4 PDEs defined the PK of immunomodulators such as IFN-β, glatiramer acetate, natalizumab, and dimethyl fumarate, which have all been evaluated in the treatment of MS. The authors used the model to explore the effects of these drugs and their combinations; the combination of the first three aforementioned drugs resulted in a decrease in initial plaque volume under specific dose combinations, emphasizing the potential efficacy of drug combinations in MS treatment.

Other modeling studies have adopted a more generalized approach to immune response dynamics (46–48). Vélez de Mendizabal et al., using a system of 6 ODEs, postulated that recurrent dynamics in autoimmunity could arise from failures in cross-regulation mechanisms between regulatory and effector T-cells (Teff), alongside stochastic events triggering the immune response (46). Their model incorporated concepts such as cross-regulation between regulatory and Teff and tissue damage. By introducing reversible and irreversible tissue damage, the model aimed at linking autoimmune activity with clinical relapses in MS patients. Simulations suggested that weakened negative feedback between effector and Treg enabled the immune system to generate characteristic RRMS dynamics without additional environmental triggers.

Another example of a generalized model has been proposed by Kannan et al. (47). It includes 4 ODEs, reflecting inflammatory and anti-inflammatory components, demyelination, and neuronal death in MS. By embedding complex control mechanisms into the equations, the authors were able to identify two key thresholds – one in the immune components and another one in the CNS – that separate distinct dynamical behaviors in the model, classifying disease subtypes *in silico* as RRMS or progressive SPMS/PPMS, based on these thresholds, once exceeded.

Expanding the ways in using mathematical tools to model detailed biological processes, Gross et al. proposed a probabilistic model to predict the differentiation and migration of lymphocyte subsets in the CNS under homeostatic and neuroinflammatory conditions (48). The 7-equation model aimed at reproducing the acquired data on the location and differentiation states of lymphocyte subsets, to provide quantitative assessments of differentiation and transmigration rates and to predict the qualitative behavior of immune-modulating therapies – thereby enabling simulation-based predictions of lymphocyte subset distributions. Validated based on data from patients with somatoform disorders, RRMS patients, and patients undergoing specific treatments, the model demonstrated accuracy in predicting differentiation stages and distribution of lymphocyte subsets under both steady-state conditions and neuroinflammatory diseases such as MS.

Finally, Broome and colleagues attempted to describe MS pathophysiology from a biochemical perspective, using a complex model capable of predicting: interactions between reactive oxygen and nitrogen species; the permeability transition pore; apoptotic factors; and eventual cell death in oligodendrocytes (49). Immune components within the model were represented only at a high level by Mφ, T-cells and several cytokines (IFN-γ, TNF-α, and IL-1), while the main focus of the model was on intracellular molecular processes. The system reflects healthy, diseased, and treated states, and allows for the identification of trigger points for disease onset and the exploration of potential drug therapies.

### 3.5 Skin

The skin is an organ constantly exposed to external factors such as injuries and infections that can disrupt the barrier function and initiate a dysregulated immune response, which may, in turn, cause chronic inflammation and autoimmunity. Despite the prevalence of autoimmune skin diseases, only two of them – alopecia areata and atopic dermatitis – have been studied using mechanistic mathematical models (50–55). Although the autoimmune nature of the latter is a matter of debate, the well-known association with multiple ADs and the presence of autoreactive T-cells and autoantibodies provide sufficient evidence for including this atopic dermatitis disorder to this review (96–99).

Atopic dermatitis (also known as eczema) is a chronic disease affecting the upper level of the skin and characterized by persistent skin inflammation. The pathogenesis of dermatitis involves epidermal barriers abnormalities, leading to heterogeneous immunological dysregulations predominantly in type 2 immunity (Th2, IL-4, IL-13, IL-31), but also including varying degrees of upregulation of the Th1 (IFN-γ), Th17 (IL-17), and Th22 axes (IL-22) (100). Tanaka and colleagues published several articles focused on the interplay between the pathogen load, immune response, and the degree of skin barrier integrity to describe recurrent dermatitis flares associated with pathogen levels surpassing certain thresholds (52–55). The model reproduces 4 types of dynamical phenotypes typically observed in patients – recovery, chronic damage, oscillations, and bi-stability – mirroring different stages of dermatitis severity. The model was applied to evaluate the effects of first-line nonspecific therapies (topical antibiotics, emollients, corticosteroids) on the transition from pathologic to healthy state. The model gradually evolved over time, for example, by introducing multiple subtypes of Th and their effector ILs (IL-4, IL-13, etc.) (55). Another significant augmentation of the modified model was the inclusion of clinical efficacy endpoints – eczema area and severity index (EASI) – which enabled a comparison of the efficacy of multiple anti-IL drugs.

Alopecia areata is an AD that causes the formation of distinct hairless patterns on the scalp or other parts of the body. According to a widely accepted hypothesis, hair loss occurs due to the collapse of the immune privilege of hair follicles under environmental factors, with subsequent migration of autoreactive lymphocytes into the hair bulb (101). A set of modeling studies by Atanaska Dobreva et al. describes the dynamics of autoreactive CD4+ and CD8+ T-cells, immune privilege guardians and IFN-γ involved in the development of alopecia, and the hair follicle growth cycles (50, 51). The developed model succeeds in capturing healthy and pathological states of a subject, highlighting the key role of IFN-γ and immune privilege guardians in disease progression. However, the uniqueness of the triggers and localness of the disease challenges the applicability of this model to other autoimmune disorders.

### 3.6 Endocrine diseases

The endocrine system is a vital regulator of biological processes, responsible for the development of the nervous system, growth, reproductive function, metabolism, and more (102). As such, autoimmune conditions associated with it usually lead to severe health complications (103).

T1DM is one such disease known from ancient times and considered fatal until the invention of recombinant insulin in the early XX^th^ century (104). The autoimmune response in T1DM is directed primarily towards β-cells in the islets of Langerhans in the pancreas, thereby depriving the organism of insulin hormone, which in turn regulates glucose uptake by tissues. Since recombinant insulin is the primary method of controlling the disease, the majority of mathematical models for this indication are focused on short- and long-term glycemic controls (105, 106). However, two models stand out, in terms of characterizing autoimmune processes relevant for the disease, and beyond insulin-glucose cross-talks (56, 57). Both models contain 5 to 6 ODEs of a highly theoretical nature, which capture the quantities of β-cells, Teff, Tregs, antigen, and other components (either Mφ or IL-2), and they allow for the exploration of the fundamental systems behavior rather than addressing questions specific to drug development. Jaberi-Douraki and colleagues highlight the impact of T-cell avidity on disease outcomes in T1DM, whereas Magombedze et al. emphasize the crucial role of the balance between Treg and auto-reactive T-cells in diabetes progression.

HT is another example of a relatively common chronic autoimmune condition, affecting the thyroid gland through an infiltration of T- and B-cells (107). Excessively stimulated Th1 cells, Th17 cells, and Tregs play important roles in the pathogenesis of HT (108). Thus, in their work, Salazar-Viedma et al. investigated the immune response mediated by Th1 and Th17 on thyrocytes (3 ODEs) (58). Interestingly, the authors also included gut microbiota into the model (a 4^th^ ODE), thereby linking autoimmunity with the function of the microbiome. Results of the modeling suggest that an imbalance in the intestinal microbiota could lead to an increase in Th17 lymphocyte activity, contributing to the development of HT.

### 3.7 Lungs

There are two mechanistic models describing autoimmune-related lung diseases, namely pulmonary sarcoidosis and IPF (59–61). It should be noted that the etiology and role of autoimmunity in the development and progression of these diseases are still unknown. However, genetic predisposition and external triggers such as infection, inorganic materials, and environmental factors are likely to lead to the development of an autoimmune response (109–112). Mφ and lymphocytes are key players involved in the initiation of the immune response in the lung, in both indications: Mφ, serving as scavenger cells, ingesting and degrading the inhaled antigenic load, initiate a typical Th1 immune response. Interestingly, both diseases are characterized by a shift from the M1 (pro-inflammatory) to the M2 (anti-inflammatory) Mφ phenotype, which is considered responsible for the progression from inflammation to interstitial fibrosis in IPF and formation of granulomas in sarcoidosis.

The first model by Hao et al. considers the progression of sarcoidosis in terms of a granuloma radius represented as a conglomerate of Mφ, Th1, Treg and Th17 cells, which affect each other through a network of cytokines and chemokines (IL-2, IL-10, IL-12, IL-13, chemokine ligand 20, IFN-γ, TNF-α and TGF-β) (59, 60). The model shows the beneficial effect of infliximab (anti-TNF-α) and other potential anti-cytokine therapies on the granuloma radius. In subsequent work, the authors investigated the progression of fibrosis in IPF by adding features unique to pulmonary tissue fibrosis, based on their previous model of kidney fibrosis (27, 61). This second model includes two Mφ phenotypes and takes into account the complex geometry of lungs through a system of PDEs, to calculate the effective interactions among model species: Mφ, epithelial and mesenchymal cells, extracellular matrix and molecular pro-fibrotic mediators (TNF-α, TGF-β, PDGF, TIMP). The authors explored the efficacy of four therapies (anti-TNF-α, anti-PDGF, anti-IL13, anti-TGF-β) and showed that only targeting TGF-β should result in a beneficial effect on pulmonary fibrosis.

### 3.8 Cardiovascular

The heart muscle, just like any other part of the body, is no exception in terms of self-stimulated inflammation and tissue damage. While rare, autoimmune myocarditis can be caused as a side effect incurred by treatment using immune checkpoint inhibitors – a large and effective class of anti-cancer therapies (113, 114). Building up on the historical research of the immune system, Van der Vegt and colleagues pioneered the modeling of checkpoint-induced autoimmune reaction, in an effort to provide an instrument that helps in balancing the benefits and risks of this type of treatment (62). The core of the developed system consists of 4 ODEs, complemented with the pharmacodynamic effects of PD-1 and CTLA-4 inhibitors as explicit functions. The model was subjected to stability and sensitivity analyses. Most importantly, model evaluation results demonstrate that the development of autoimmune myocarditis under treatment with high doses of nivolumab or ipilimumab is practically inevitable, depending on individual patient characteristics reflected in the values of several key model parameters.

### 3.9 Generalized models of autoimmune processes

Mathematical modeling, by implication, is a transformation of empirical knowledge into a system of cause-consequence relationships using the universal language of mathematics, theoretically applicable to any object or phenomenon. As such, not all models are necessarily associated with specific diseases but rather focus on the general behavior of immune response components potentially leading to autoimmune disorders. Although mostly qualitative, inferences derived from these models significantly broaden our understanding of interactions within the system of interest and provide a companion tool for further experimental research.

A notable early example of such a mathematical system is the ICMODEL developed by Martha Head et al. (63). This model represents a theoretical interpretation of a mechanism for IC formation, opsonization, and clearance. It consists of 3 sub-modules: (1) production of antibodies and their interaction with antigen (*i.e.*, IC formation); (2) opsonization and clearance of IC; (3) tissue damage and antigen release. The model was explored for consistency with the known behavior of the immune system, evaluated for stability, and then applied to identify processes leading to a substantial rise or oscillations in pathogenic IC concentrations (*e.g.*, impaired antigen efflux, Fc-γ-mediated phagocytosis, complement synthesis). Such a model may be beneficial in understanding the cyclical course of various autoimmune disorders.

Later, Arazi and Neumann developed a more parsimonious model focused on the same spectrum of processes as the ICMODEL; it consists of 3 ODEs reflecting the quantities of autoreactive B-cells, autoantigen, and IC (64). Thorough steady-state, hysteresis, and parameter sensitivity analyses, as well as an evaluation of the different functional relationships was performed, to demonstrate that a positive feedback loop between IC and tissue damage would not be sufficient to drive the system to a pathological state - thereby confirming that the clearance rates of ICs and self-antigens are one of the key factors triggering a clinically impactful autoimmune response.

Another example, similar in principle to the work by Arazi and Neumann, is a minimalistic ODE model by Iwami and colleagues that includes 3 dependent variables: population size of target cells, damaged cells, and immune cells (65). The authors proposed several functional relationships (linear and non-linear) of target cell growth and immune response followed by a stability analysis, to describe mathematically different states of immune tolerance/dormancy and repeated flare-ups.

Further modeling work can be found in a generalized model by Khailaie et al., in a series of studies dedicated to the investigation of T-cell activation (66). However, this paper is centered around disturbances in the delicate balance between Tcon and Tregs leading to autoimmunity. The authors methodically tested different models, starting from the simplest structure with 2 ODEs describing the dynamics of activated T-cells and IL-2 and ending with pathways of Tregs and Tcon renewal rates, activation, proliferation, and reciprocal influences. They derived an antigen-stimulation threshold (*i.e.*, the proliferation rate of activated T-cells depending on the avidity to an antigen) that defines three scenarios of immune response: a complete lack of a response; a first peak followed by complete clearance of the antigen; or the failure of such, which would result in chronic persistence of the antigen, and is primarily defined by the renewal rate of naïve T-cells and resting Tregs.

The balance of T-cells, although Th1 and Th2 instead of Tcon and Tregs, is the subject of another model-based analysis, performed by Louzoun et al. (67). This work, published in 2001, thoroughly explores mathematical system that includes 5 cell types (Th1, Th2, Mφ, naïve T-cells, and pooled Teff and Treg) and 3 types of cytokines, aggregated by associated cells (Th1, Th2, or Mφ), to characterize the transition from healthy condition (designated as Th2 steady-state) to autoimmunity (designated as Th1 steady-state). Although the rationale behind the model is based on the preclinical observations (*i.e.*, insulin-dependent diabetes and experimental autoimmune encephalomyelitis mouse models), proposed model is positioned as qualitative investigation of Th1-mediated autoimmune diseases.

Another aspect of autoimmune disease onset, namely immune response to self-antigen caused by cross-reactivity to viral antigen, was investigated by Hara and Iwasa using a model in 4 ODEs, which reflects the kinetics of Th-cells (reactive to self-antigen or viral infection), viral antigen, and memory T-cells (68). As in the case of previous qualitative model, characterization of general patterns in the dynamics of the system through sensitivity analysis, including the evaluation of autoimmune response relative to the different modes of cross-immunity, rather than qualitative predictions of the treatment-related effects was the focus of this research.

Continuing the thread of qualitative models, the study by Machado Ramos et al. considers three cell populations associated with autoimmune response – self-APCs, self-reactive T-cells, and immunosuppressive cells (69). The model was developed using a combination of integro-differential equations and ODEs to track the total number of cells within each population and their respective functional states and tested for robustness. Sensitivity analysis of the model highlights the critical roles of proliferation of immunosuppressive cell, destruction of self-APC and self-reactive T-cell, and the tolerance of self-reactive T-cells to self-antigens in regulating autoimmune responses effectively.

The last piece of modeling work evaluated in this section is devoted to the mathematical description of two generic, inter-dependent immune cell populations, mutually regulating one another *via* cytokine production (70). The interactions between the two cytokine production profiles reflect the point of a synergistic balance, where both cell populations reach equilibrium. An exhaustive screening of possible parameter values revealed that an AD may occur when the alteration of the feedback between immune cells results in hyper-induced homeostasis or a loss of stability in the system, leading to oscillations or trigger-based inflammatory disease phenotypes.

## 4 Discussion

Mathematical modeling in drug development is an essential tool necessary to transform heterogeneous experimental data from numerous sources into a system of quantitative knowledge, with an ultimate goal of facilitating the delivery of innovative therapies to patients. The indispensability of this tool is especially noticeable in therapeutic areas such as ADs, with non-trivial diversity in pathogenesis, etiology, manifestations, and the associated plethora of unanswered questions related to drug development. Among modeling methods, empirical PK/PD models first introduced in the 1970s and focused on the direct assessment of the dose-concentration-effect relationship can be considered as the most established and frequently used models (115, 116). In ADs, this type of research has been pioneered by Brooks et al., with a model-based investigation of the clinical effects of concomitant use of indomethacin and furosemide in RA patients (117). Since then, the number of publications that could be attributed towards PK/PD modeling in ADs grew almost exponentially (**Figure 1A**). However, the utility of empirical models can be limited by their simplicity, whereas multiple drug R&D questions, such as target selection, animal-to-human translation, and others, require an in-depth analysis of the underlying biological mechanisms (16, 17).

This is where mechanistic models may come into play. These models represent a slice of knowledge in selected disease areas, and, therefore, first understanding the scope of a given endeavor in this field is important, to focus the efforts of subsequent research – which is the main purpose of this review. In our systematic search and curation, we identified 38 mechanistic models of ADs across 47 publications. While it is challenging to define the fine line between mechanistic and non-mechanistic models, in this paper, we attributed models to the former category if they contained a description of autoimmune processes at any level of generalization, regardless of the mathematical method used or the number of equations featured. It is worth noting that the majority of the identified models (>70%) are expressed as ODEs, similarly to most systems pharmacology models, with a minimum of 3 equations being featured in their structure (**Figure 1C**) (118). In addition to ODE models, two groups of models featuring PDEs were found, with a distinct focus on tissue remodeling: a set of publications by Wenrui Hao and colleagues (27, 28, 60, 61), examining kidney and lung fibrosis, and models of plaque growth in MS and cartilage degradation in RA, by Moise and Friedman (31, 45). A Boolean model was developed by Ruiz-Cerdá and colleagues, to describe the antigen presentation process in lupus (24), and another Boolean model with subsequent ODE extensions, by Balbas-Martinez et al., was identified for IBD (41, 42). Finally, a unique probabilistic (Markov jump processes) model by Catharina Gross and colleagues was developed, to capture the differentiation and migration of lymphocyte subsets to CNS (48).

The number of publications related to mechanistic modeling is overall markedly smaller as compared to PK/PD modeling studies, with a slower and shallower increase over time (**Figure 1A**), which could be explained by the time and efforts, as well as the high expertise in immunology required, to build such models. Interestingly, while the search contained more than 180 terms for different ADs (see **Supplementary Materials**), only models for 13 disease-specific were identified by the search, with models for 2 systemic conditions (SLE and RA) and 11 for organ-specific indications (**Figure 1B**). Hence, the mechanisms for more than 150 other ADs, such as psoriasis, systemic sclerosis, involvement of the urinary system and Sjogren’s syndrome, are not covered by the currently available mathematical modeling literature, and thus present a high opportunity for further research. However, the biological entities embedded in the identified models are all linked to the immune response and are thus expected to overlap in these other ADs. Indeed, the analysis of variables and their interactions among currently available models revealed several common clusters (**Figure 2**).

Approximately 1/3 of the models included antigens, in a broad sense (*e.g.*, pathogen load, commensal bacteria, damaged tissue), as a dependent variable, which is not surprising considering ADs are characterized by responses to self-antigen or may be triggered *via* external microorganisms. A similar frequency of occurrence in the models was observed for Tregs, important gatekeepers of the immune system that inhibit the activation and expansion of Th, cytotoxic T-cells and B-cells. Their role has been extensively described in SLE, RA and MS (119). Overall, most of the models explored T-cell-mediated immune response with an emphasis on Th, with a different degree of generalization (*i.e.*, either by pooling different subtypes of cells into generic variables or by considering them individually). In contrast, autoreactive cytotoxic T lymphocytes characterized by cytolytic activities against target cells are under-represented in the models and are often lumped together into effector T-cell variables. Other types of lymphocytes are modeled only in isolated cases, even in ADs classified as B-cell-mediated or antibody-driven pathologies, such as SLE (120).

In addition to featuring immune cells, the models cover a wide range of pro- and anti-inflammatory ILs as a critical part of cross-regulation, proliferation, and polarization of Th subtypes. For example, 6 out of 36 models incorporate the connection between IL-2 and Tregs, as well as IFN-γ and Th1. Furthermore, cytokines with well-established roles in AD progression (*e.g.*, TNF-α, IL-6, IL-23, etc.) and respective therapeutic interventions are also frequently included in the models. Despite this, none of them contain type I IFN (*e.g.*, IFN-α or IFN-β), although its substantial role has been highlighted in several ADs, including SLE, where multiple approved or developing therapies are designed to block the type I IFN-inducible pathway of inflammation.

Lastly, tissue damage or fibrosis is also frequently modeled, as a final outcome of AD progression. In **Figure 2**, tissue scarring is associated with a cluster of Mφ, GM-CSF, and a lumped term “fibrosis” that includes MMPs, elements of the extracellular matrix, fibroblasts, and several growth factors (*e.g.*, TGF-β, FGF, PDGF). Tissue damage is either modeled as a generic variable or through a number of organ-specific cells, *e.g.*, thyrocytes in HT. It is worth noting that the main mechanisms of tissue damage, consisting of cytotoxic activity of autoreactive CD8+ T-cells and IC-related activation of complement system, are missing in these models, with few exceptions that link IC to cell death.

While mathematical modeling is an essential tool to support decision-making, the practical value of a particular model for drug development is defined by the adherence to good modeling practices and is inherently limited by the underlying assumptions (121). The latter is directly tied to the data used for model development and calibration. About 40% of the identified models were built around clinical data only, whereas practically 29% of models additionally involved animal data, when information in human was lacking or scarce (**Figure 1D**). Mixing such different types of data may be a sensitive subject to perform model inference, and it calls for further verification of the similarities in immune mechanisms across species, as a distinct direction for future analyses (122, 123). Among good modeling practices, several key components could be named, *e.g.*, extensive model calibration and evaluation, including identifiability analyses, external validation, sensitivity analyses, and more (124). Several models, *e.g.*, the works of Gao et al. (29), Nakada and Mager (32), Rogers et al. (39, 40), and Miyano et al. (55) feature a broad array of these technical model-based analyses. Furthermore, these models attempt to incorporate subject inter-individual variability through the means of generating realistic virtual populations; they also consider realistic PK models of the compounds, driving the effects, which greatly expands the applicability of such models to real-world scenarios. These models may be considered as an exception, since the remaining models discussed here do not rely on parameter identifiability, extensive model diagnostics or advanced simulations. In fact, nearly a quarter of all models considered in this review operate as a system without a direct link to the observed time series of laboratory measurements (**Figure 1D**) and do not make use of external validation techniques (**Table 1**). This may be explained by differences in the relevant questions that motivate the development of such models. For example, modeling studies classified as describing general autoimmune reactions as well as several others are built to explore mathematical systems with specific behaviors, through an analysis of stability and phase portraits (*e.g.*, to describe periodical nature of flares) (64, 66). Likewise, many models are primarily developed to perform a sensitivity analysis, with parameter values assumed or fine-tuned manually (65, 70). As a result, the potential impact of such models can be limited to mechanistic hypothesis generation and target validation. Because of that reason, our search was restricted to the PubMed database, which specifically indexes biomedical and life sciences journals. As such, it should be mentioned that the total number of published model-based analyses of autoimmune processes exceeds those discussed in detail in this review e.g., (125–131). However, the focus of these studies lies primarily in the analysis of specific patterns of mathematical systems, their phase trajectories, steady-states, etc., rarely informed by the large quantities of clinical data and thereby, while providing important insights into theoretical behavior of the immune system, have limited practical applicability in model-informed drug development.

In summary, the applicability of current mechanistic models of ADs in drug development could be limited by several factors. Firstly, the lack of evaluation of model performance against the observed clinical data, including the assessment of uncertainty in model parameters and the validation against independent data, results in qualitative predictions of system behavior rather than quantitative ones. Secondly, most existing models do not incorporate PK submodules to describe drug exposure in target compartments over time. Furthermore, some components of the immune system are under-represented or simply are lacking in the available models. For instance, current treatment options for SLE are focused primarily in three areas: disrupting type I IFN-mediated inflammation, targeting B-cell response, and inhibiting intracellular signaling; none of which are among the modeled immune components (15). Finally, to provide insight into an actual clinical benefit, the models should strive to incorporate clinical endpoints. Currently available models are built upon inferences around surrogate biomarkers or generic variables (*e.g.*, tissue damage or fibrosis), with a single exception by Miyano et al. (55), where the EASI endpoint was described as a function of skin barrier integrity and infiltrated pathogens.

This analysis follows the requirements of a robust systematic review, with documented sources and search queries, and, to our knowledge, is a unique venture to comprehensively identify and categorize existing mechanistic models of ADs. It should not be viewed as an objective effort to qualify the formidable effort that each of the analyzed models represents, as the value of modeling is always relative to its aim. Rather, it is an attempt to draw a landscape of the ever-evolving quantitative knowledge on ADs in the form of mathematical models to define the most desirable paths of moving forward in relation to drug development. In this context, the amount of robust QSP models, with extensive model calibration and evaluation and built on clinical data, while maintaining a delicate balance between the available data and model complexity, is significantly lacking in the considered disease domain. This gap is further broadened by the emergence of new therapeutic modalities (*e.g.*, chimeric antigen receptor T-cell), not yet covered by these existing models (132). Taken together, these results highlight the necessity and aspiration to enhance proactiveness and efforts in mechanistic quantitative analyses in the intricate therapeutic area of ADs, to facilitate the development of novel therapeutics.

## Author contributions

YU: Data curation, Formal Analysis, Investigation, Methodology, Software, Visualization, Writing – original draft. AN: Formal Analysis, Investigation, Writing – original draft. CL: Formal Analysis, Investigation, Methodology, Visualization, Writing – original draft. GH: Writing – review & editing. KP: Funding acquisition, Resources, Writing – review & editing. VS: Conceptualization, Methodology, Project administration, Supervision, Writing – review & editing. AV: Conceptualization, Funding acquisition, Methodology, Project administration, Supervision, Validation, Writing – original draft, Writing – review & editing.
