# Corrigendum: Mathematical modeling in autoimmune diseases: from theory to clinical application

**DOI:** 10.3389/fimmu.2025.1557929

**Published:** 2025-01-30

**Authors:** Yaroslav Ugolkov, Antonina Nikitich, Cristina Leon, Gabriel Helmlinger, Kirill Peskov, Victor Sokolov, Alina Volkova

**Affiliations:** ^1^ Research Center of Model-Informed Drug Development, Ivan Mikhaylovich (I.M.) Sechenov First Moscow State Medical University, Moscow, Russia; ^2^ Marchuk Institute of Numerical Mathematics of the Russian Academy of Sciences (RAS), Moscow, Russia; ^3^ Faculty of Bioengineering and Bioinformatics, Lomonosov Moscow State University, Moscow, Russia; ^4^ Modeling and Simulation Decisions FZ - LLC, Dubai, United Arab Emirates; ^5^ Biorchestra Co., Ltd., Cambridge, MA, United States; ^6^ Sirius University of Science and Technology, Sirius, Russia

**Keywords:** autoimmune diseases, mathematical modeling, quantitative systems pharmacology, model-informed drug development, immune system modeling

In the published article, there was an error regarding the affiliation for Yaroslav Ugolkov. As well as having affiliation(s) 1, 2, they should also have Faculty of Bioengineering and Bioinformatics, Lomonosov Moscow State University, Moscow, Russia.

The authors apologize for this error and state that this does not change the scientific conclusions of the article in any way. The original article has been updated.

