# Supplementary material for: Mathematical modeling in autoimmune diseases: from theory to clinical application
Source: Front Immunol. 2024 Mar 14;15:1371620. doi: 10.3389/fimmu.2024.1371620 (PMC10973044; doi:10.3389/fimmu.2024.1371620)
Supplement: Supplementary file 1 [file DataSheet_1.docx]

***Supplementary Material***

**Mathematical modeling in autoimmune diseases: from theory to clinical application**

Yaroslav Ugolkov, Antonina Nikitich, Cristina Leon, Gabriel Helmlinger, Kirill Peskov, Victor Sokolov and Alina Volkova^*^

*Correspondence: Alina Volkova, [alina.volkova@msdecisions.tech](mailto:alina.volkova@msdecisions.tech)

# Supplementary Methods

**PubMed query for the search of mechanistic mathematical models in autoimmune diseases:**

((autoimmune diseas*[Title/Abstract]) OR ( autoimmun*[Title/Abstract]) OR ( autoinflammatory[Title/Abstract] ) OR ( Acquired aplastic anemia[Title/Abstract]) OR ( Acquired hemophilia[Title/Abstract]) OR ( Acromegaly[Title/Abstract]) OR ( Addison's disease[Title/Abstract]) OR ( Agammaglobulinemia[Title/Abstract]) OR ( Alopecia areata[Title/Abstract]) OR ( Ankylosing Spondylitis[Title/Abstract]) OR ( Anti-NMDA receptor encephalitis[Title/Abstract]) OR ( Antiphospholipid syndrome [Title/Abstract]) OR ( Arteriosclerosis[Title/Abstract]) OR ( Asherson's syndrome[Title/Abstract]) OR ( Atopic Dermatitis[Title/Abstract]) OR ( Autoimmune Addison’s disease [Title/Abstract]) OR ( Autoimmune autonomic ganglionopathy [Title/Abstract]) OR ( Autoimmune encephalitis[Title/Abstract]) OR ( Autoimmune gastritis[Title/Abstract]) OR ( Autoimmune hemolytic anemia[Title/Abstract]) OR ( Autoimmune hepatitis[Title/Abstract]) OR ( Autoimmune hyperlipidemia[Title/Abstract]) OR ( autoimmune hypophysitis[Title/Abstract]) OR ( Autoimmune inner ear disease [Title/Abstract]) OR ( Autoimmune lymphoproliferative syndrome [Title/Abstract]) OR ( Autoimmune myelofibrosis[Title/Abstract]) OR ( Autoimmune myocarditis[Title/Abstract]) OR ( Autoimmune oophoritis[Title/Abstract]) OR ( Autoimmune pancreatitis [Title/Abstract]) OR ( Autoimmune polyglandular syndromes[Title/Abstract]) OR ( Autoimmune progesterone dermatitis[Title/Abstract]) OR ( Autoimmune retinopathy [Title/Abstract]) OR ( Autoimmune sudden sensorineural hearing loss [Title/Abstract]) OR ( autoimmune thrombocytopenic purpura [Title/Abstract]) OR ( autoimmune thyroiditis [Title/Abstract]) OR ( Balo disease[Title/Abstract]) OR ( Behçet’s disease[Title/Abstract]) OR ( Behcet's disease[Title/Abstract]) OR ( Berger's disease [Title/Abstract]) OR ( Birdshot chorioretinopathy [Title/Abstract]) OR ( Bullous pemphigoid[Title/Abstract]) OR ( Castleman disease[Title/Abstract]) OR ( catastrophic antiphospholipid syndrome [Title/Abstract]) OR ( Celiac disease[Title/Abstract]) OR ( Chagas disease[Title/Abstract]) OR ( Chronic active hepatitis [Title/Abstract]) OR ( Chronic autoimmune urticaria[Title/Abstract]) OR ( Chronic inflammatory demyelinating polyneuropathy [Title/Abstract]) OR ( chronic lymphocytic thyroiditis [Title/Abstract]) OR ( Churg-Strauss syndrome[Title/Abstract]) OR ( Coeliac[Title/Abstract]) OR ( Cogan’s syndrome[Title/Abstract]) OR ( Cold agglutinin disease[Title/Abstract]) OR ( Complex regional pain syndrome [Title/Abstract]) OR ( CREST syndrome[Title/Abstract]) OR ( Crohn’s disease [Title/Abstract]) OR ( Cronkhite-Canada syndrome [Title/Abstract]) OR ( Cryptogenic organizing pneumonia [Title/Abstract]) OR ( Dermatitis herpetiformis[Title/Abstract]) OR ( Dermatomyositis[Title/Abstract]) OR ( Devic's disease[Title/Abstract]) OR ( Discoid lupus[Title/Abstract]) OR ( Dressler’s syndrome [Title/Abstract]) OR ( Eczema[Title/Abstract]) OR ( Endometriosis[Title/Abstract]) OR ( Eosinophilic esophagitis[Title/Abstract]) OR ( Eosinophilic fasciitis[Title/Abstract]) OR ( Erythema nodosum[Title/Abstract]) OR ( Essential mixed cryoglobulinemia[Title/Abstract]) OR ( Evans syndrome[Title/Abstract]) OR ( Fibrosing alveolitis[Title/Abstract]) OR ( Giant cell arteritis[Title/Abstract]) OR ( Giant Cell Myocarditis[Title/Abstract]) OR ( Glomerulonephritis[Title/Abstract]) OR ( Goodpasture’s syndrome[Title/Abstract]) OR ( Granulomatosis with polyangiitis [Title/Abstract]) OR ( Graves disease[Title/Abstract]) OR ( Guillain-Barré syndrome [Title/Abstract]) OR ( Hashimoto thyroiditis[Title/Abstract]) OR ( Hemifacial atrophy [Title/Abstract]) OR ( Henoch-Schönlein purpura[Title/Abstract]) OR ( Hidradenitis suppurativa[Title/Abstract]) OR ( Horton’s disease [Title/Abstract]) OR ( Hurst’s disease[Title/Abstract]) OR ( Hypogammaglobulinemia[Title/Abstract]) OR ( Idiopathic pulmonary fibrosis [Title/Abstract]) OR ( Idiopathic thrombocytopenia purpura[Title/Abstract]) OR ( IgA nephropathy [Title/Abstract]) OR ( IgA vasculitis [Title/Abstract]) OR ( Henoch Schönlein purpura [Title/Abstract]) OR ( IgG4-related sclerosing disease [Title/Abstract]) OR ( Immune thrombocytopenia [Title/Abstract]) OR ( Immune-mediated necrotizing myopathy [Title/Abstract]) OR ( Inclusion body myositis[Title/Abstract]) OR ( Inflammatory bowel disease[Title/Abstract]) OR ( Interstitial cystitis[Title/Abstract]) OR ( Juvenile idiopathic arthritis [Title/Abstract]) OR ( Kawasaki disease[Title/Abstract]) OR ( Lambert-Eaton myasthenic syndrome [Title/Abstract]) OR ( Leukocytoclastic vasculitis[Title/Abstract]) OR ( Lichen planopilaris [Title/Abstract]) OR ( Lichen planus [Title/Abstract]) OR ( Lichen sclerosus[Title/Abstract]) OR ( Ligneous conjunctivitis[Title/Abstract]) OR ( linear IgA bullous dermatosis [Title/Abstract]) OR ( Linear IgA disease [Title/Abstract]) OR ( Lupus nephritis[Title/Abstract]) OR ( Lymphocytic colitis[Title/Abstract]) OR ( Ménière’s disease[Title/Abstract]) OR ( Microscopic polyangiitis [Title/Abstract]) OR ( Mixed connective tissue disease [Title/Abstract]) OR ( Mooren’s ulcer[Title/Abstract]) OR ( Mucha-Habermann disease[Title/Abstract]) OR ( Multifocal motor neuropathy[Title/Abstract]) OR ( Multiple sclerosis [Title/Abstract]) OR ( Myalgic encephalomyelitis [Title/Abstract]) OR ( Myasthenia gravis [Title/Abstract]) OR ( Myocarditis[Title/Abstract]) OR ( Myositis [Title/Abstract]) OR ( Narcolepsy[Title/Abstract]) OR ( Ocular cicatricial pemphigoid[Title/Abstract]) OR ( Opsoclonus-myoclonus syndrome [Title/Abstract]) OR ( Palindromic rheumatism[Title/Abstract]) OR ( Palmoplantar Pustulosis[Title/Abstract]) OR ( Paraneoplastic cerebellar degeneration[Title/Abstract]) OR ( Paraneoplastic pemphigus[Title/Abstract]) OR ( Paroxysmal nocturnal hemoglobinuria [Title/Abstract]) OR ( Parry-Romberg syndrome [Title/Abstract]) OR ( Parsonage-Turner syndrome[Title/Abstract]) OR ( Pemphigus foliaceus[Title/Abstract]) OR ( Pemphigus gestationis[Title/Abstract]) OR ( Pemphigus vulgaris[Title/Abstract]) OR ( Peripheral uveitis[Title/Abstract]) OR ( Pernicious anemia [Title/Abstract]) OR ( POEMS syndrome[Title/Abstract]) OR ( Polyarteritis nodosa[Title/Abstract]) OR ( Polymyalgia rheumatica[Title/Abstract]) OR ( Polymyositis[Title/Abstract]) OR ( postpericardiotomy syndrome[Title/Abstract]) OR ( Postural orthostatic tachycardia syndrome [Title/Abstract]) OR ( primary biliary cholangitis[Title/Abstract]) OR ( Primary biliary cirrhosis [Title/Abstract]) OR ( Primary sclerosing cholangitis [Title/Abstract]) OR ( Primary systemic vasculitisa [Title/Abstract]) OR ( Progressive facial hemiatrophy[Title/Abstract]) OR ( Psoriasis[Title/Abstract]) OR ( Psoriatic arthritis[Title/Abstract]) OR ( Pulmonary fibrosis[Title/Abstract]) OR ( Pure red cell aplasia [Title/Abstract]) OR ( Pyoderma gangrenosum[Title/Abstract]) OR ( Rasmussen's encephalitis[Title/Abstract]) OR ( Raynaud’s syndrome[Title/Abstract]) OR ( Reflex sympathetic dystrophy syndrome [Title/Abstract]) OR ( Reiter’s syndrome[Title/Abstract]) OR ( Relapsing polychondritis[Title/Abstract]) OR ( Restless leg syndrome [Title/Abstract]) OR ( Rheumatic fever[Title/Abstract]) OR ( Rheumatic fever and Rheumatic [Title/Abstract]) OR ( Rheumatoid arthritis[Title/Abstract]) OR ( Sarcoidosis[Title/Abstract]) OR ( Schmidt syndrome[Title/Abstract]) OR ( Scleritis[Title/Abstract]) OR ( Scleroderma [Title/Abstract]) OR ( Sclerosing Mesenteritis[Title/Abstract]) OR ( Serpiginous choroidopathy[Title/Abstract]) OR ( Sjögren’s syndrome[Title/Abstract]) OR ( Small fiber sensory neuropathy[Title/Abstract]) OR ( Stiff person syndrome [Title/Abstract]) OR ( Subacute bacterial endocarditis [Title/Abstract]) OR ( Subacute cutaneous lupus[Title/Abstract]) OR ( Susac syndrome[Title/Abstract]) OR ( Sydenham's chorea[Title/Abstract]) OR ( Sympathetic ophthalmia[Title/Abstract]) OR ( Systemic lupus erythematosus[Title/Abstract]) OR ( Systemic sclerosis[Title/Abstract]) OR ( Takayasu’s arteritis [Title/Abstract]) OR ( temporal arteritis [Title/Abstract]) OR ( Testicular autoimmunity [Title/Abstract]) OR ( Thyroiditis [Title/Abstract]) OR ( Tolosa-Hunt syndrome[Title/Abstract]) OR ( Transverse myelitis [Title/Abstract]) OR ( Tubulointerstitial nephritis uveitis syndrome [Title/Abstract]) OR ( Type 1 diabetes[Title/Abstract]) OR ( Ulcerative colitis [Title/Abstract]) OR ( Undifferentiated connective tissue disease [Title/Abstract]) OR ( Uveitis [Title/Abstract]) OR ( Vasculitis[Title/Abstract]) OR ( VEXAS Syndrome[Title/Abstract]) OR ( Vitiligo[Title/Abstract]) OR ( Vogt-Koyanagi-Harada syndrome [Title/Abstract]) OR ( Wegener’s granulomatosis[Title/Abstract]) OR ( Willis-Ekbom disease[Title/Abstract])) AND ( (((mathematical model*[Title/Abstract]) OR (computational model[Title/Abstract]) OR (kinetic model[Title/Abstract])) AND ((mechanistic* [Title/Abstract]) OR ( system*[Title/Abstract]) OR ( dynamic*[Title/Abstract]) OR ( progress* [Title/Abstract]) OR (modeling[Title/Abstract])) ) OR (mechanistic model*[Title/Abstract]) OR (Physiologically based [Title/Abstract]) OR ( Quantitative systems pharmacology [Title/Abstract]) OR ( QSP[Title/Abstract])) AND (english[Filter]) NOT (("clinical trial"[Publication Type]) OR ("clinical trial protocol"[Publication Type]) OR ("clinical trial, phase i"[Publication Type]) OR ("clinical trial, phase ii"[Publication Type]) OR ("clinical trial, phase iii"[Publication Type]) OR ("clinical trial, phase iv"[Publication Type]) OR (dog[Title/Abstract]) OR (dogs[Title/Abstract]) OR (Chagas [Title/Abstract]) OR ( "review"[Publication Type]) OR ( "systematic review"[Publication Type]) OR ( "meta analysis"[Publication Type]))

**PubMed query for the search of pharmacokinetics and pharmacodynamics mathematical models in autoimmune diseases:**

((autoimmune diseas*[Title/Abstract]) OR ( autoimmun*[Title/Abstract]) OR ( autoinflammatory[Title/Abstract] ) OR ( Acquired aplastic anemia[Title/Abstract]) OR ( Acquired hemophilia[Title/Abstract]) OR ( Acromegaly[Title/Abstract]) OR ( Addison's disease[Title/Abstract]) OR ( Agammaglobulinemia[Title/Abstract]) OR ( Alopecia areata[Title/Abstract]) OR ( Ankylosing Spondylitis[Title/Abstract]) OR ( Anti-NMDA receptor encephalitis[Title/Abstract]) OR ( Antiphospholipid syndrome [Title/Abstract]) OR ( Arteriosclerosis[Title/Abstract]) OR ( Asherson's syndrome[Title/Abstract]) OR ( Atopic Dermatitis[Title/Abstract]) OR ( Autoimmune Addison’s disease [Title/Abstract]) OR ( Autoimmune autonomic ganglionopathy [Title/Abstract]) OR ( Autoimmune encephalitis[Title/Abstract]) OR ( Autoimmune gastritis[Title/Abstract]) OR ( Autoimmune hemolytic anemia[Title/Abstract]) OR ( Autoimmune hepatitis[Title/Abstract]) OR ( Autoimmune hyperlipidemia[Title/Abstract]) OR ( autoimmune hypophysitis[Title/Abstract]) OR ( Autoimmune inner ear disease [Title/Abstract]) OR ( Autoimmune lymphoproliferative syndrome [Title/Abstract]) OR ( Autoimmune myelofibrosis[Title/Abstract]) OR ( Autoimmune myocarditis[Title/Abstract]) OR ( Autoimmune oophoritis[Title/Abstract]) OR ( Autoimmune pancreatitis [Title/Abstract]) OR ( Autoimmune polyglandular syndromes[Title/Abstract]) OR ( Autoimmune progesterone dermatitis[Title/Abstract]) OR ( Autoimmune retinopathy [Title/Abstract]) OR ( Autoimmune sudden sensorineural hearing loss [Title/Abstract]) OR ( autoimmune thrombocytopenic purpura [Title/Abstract]) OR ( autoimmune thyroiditis [Title/Abstract]) OR ( Balo disease[Title/Abstract]) OR ( Behçet’s disease[Title/Abstract]) OR ( Behcet's disease[Title/Abstract]) OR ( Berger's disease [Title/Abstract]) OR ( Birdshot chorioretinopathy [Title/Abstract]) OR ( Bullous pemphigoid[Title/Abstract]) OR ( Castleman disease[Title/Abstract]) OR ( catastrophic antiphospholipid syndrome [Title/Abstract]) OR ( Celiac disease[Title/Abstract]) OR ( Chagas disease[Title/Abstract]) OR ( Chronic active hepatitis [Title/Abstract]) OR ( Chronic autoimmune urticaria[Title/Abstract]) OR ( Chronic inflammatory demyelinating polyneuropathy [Title/Abstract]) OR ( chronic lymphocytic thyroiditis [Title/Abstract]) OR ( Churg-Strauss syndrome[Title/Abstract]) OR ( Coeliac[Title/Abstract]) OR ( Cogan’s syndrome[Title/Abstract]) OR ( Cold agglutinin disease[Title/Abstract]) OR ( Complex regional pain syndrome [Title/Abstract]) OR ( CREST syndrome[Title/Abstract]) OR ( Crohn’s disease [Title/Abstract]) OR ( Cronkhite-Canada syndrome [Title/Abstract]) OR ( Cryptogenic organizing pneumonia [Title/Abstract]) OR ( Dermatitis herpetiformis[Title/Abstract]) OR ( Dermatomyositis[Title/Abstract]) OR ( Devic's disease[Title/Abstract]) OR ( Discoid lupus[Title/Abstract]) OR ( Dressler’s syndrome [Title/Abstract]) OR ( Eczema[Title/Abstract]) OR ( Endometriosis[Title/Abstract]) OR ( Eosinophilic esophagitis[Title/Abstract]) OR ( Eosinophilic fasciitis[Title/Abstract]) OR ( Erythema nodosum[Title/Abstract]) OR ( Essential mixed cryoglobulinemia[Title/Abstract]) OR ( Evans syndrome[Title/Abstract]) OR ( Fibrosing alveolitis[Title/Abstract]) OR ( Giant cell arteritis[Title/Abstract]) OR ( Giant Cell Myocarditis[Title/Abstract]) OR ( Glomerulonephritis[Title/Abstract]) OR ( Goodpasture’s syndrome[Title/Abstract]) OR ( Granulomatosis with polyangiitis [Title/Abstract]) OR ( Graves disease[Title/Abstract]) OR ( Guillain-Barré syndrome [Title/Abstract]) OR ( Hashimoto thyroiditis[Title/Abstract]) OR ( Hemifacial atrophy [Title/Abstract]) OR ( Henoch-Schönlein purpura[Title/Abstract]) OR ( Hidradenitis suppurativa[Title/Abstract]) OR ( Horton’s disease [Title/Abstract]) OR ( Hurst’s disease[Title/Abstract]) OR ( Hypogammaglobulinemia[Title/Abstract]) OR ( Idiopathic pulmonary fibrosis [Title/Abstract]) OR ( Idiopathic thrombocytopenia purpura[Title/Abstract]) OR ( IgA nephropathy [Title/Abstract]) OR ( IgA vasculitis [Title/Abstract]) OR ( Henoch Schönlein purpura [Title/Abstract]) OR ( IgG4-related sclerosing disease [Title/Abstract]) OR ( Immune thrombocytopenia [Title/Abstract]) OR ( Immune-mediated necrotizing myopathy [Title/Abstract]) OR ( Inclusion body myositis[Title/Abstract]) OR ( Inflammatory bowel disease[Title/Abstract]) OR ( Interstitial cystitis[Title/Abstract]) OR ( Juvenile idiopathic arthritis [Title/Abstract]) OR ( Kawasaki disease[Title/Abstract]) OR ( Lambert-Eaton myasthenic syndrome [Title/Abstract]) OR ( Leukocytoclastic vasculitis[Title/Abstract]) OR ( Lichen planopilaris [Title/Abstract]) OR ( Lichen planus [Title/Abstract]) OR ( Lichen sclerosus[Title/Abstract]) OR ( Ligneous conjunctivitis[Title/Abstract]) OR ( linear IgA bullous dermatosis [Title/Abstract]) OR ( Linear IgA disease [Title/Abstract]) OR ( Lupus nephritis[Title/Abstract]) OR ( Lymphocytic colitis[Title/Abstract]) OR ( Ménière’s disease[Title/Abstract]) OR ( Microscopic polyangiitis [Title/Abstract]) OR ( Mixed connective tissue disease [Title/Abstract]) OR ( Mooren’s ulcer[Title/Abstract]) OR ( Mucha-Habermann disease[Title/Abstract]) OR ( Multifocal motor neuropathy[Title/Abstract]) OR ( Multiple sclerosis [Title/Abstract]) OR ( Myalgic encephalomyelitis [Title/Abstract]) OR ( Myasthenia gravis [Title/Abstract]) OR ( Myocarditis[Title/Abstract]) OR ( Myositis [Title/Abstract]) OR ( Narcolepsy[Title/Abstract]) OR ( Ocular cicatricial pemphigoid[Title/Abstract]) OR ( Opsoclonus-myoclonus syndrome [Title/Abstract]) OR ( Palindromic rheumatism[Title/Abstract]) OR ( Palmoplantar Pustulosis[Title/Abstract]) OR ( Paraneoplastic cerebellar degeneration[Title/Abstract]) OR ( Paraneoplastic pemphigus[Title/Abstract]) OR ( Paroxysmal nocturnal hemoglobinuria [Title/Abstract]) OR ( Parry-Romberg syndrome [Title/Abstract]) OR ( Parsonage-Turner syndrome[Title/Abstract]) OR ( Pemphigus foliaceus[Title/Abstract]) OR ( Pemphigus gestationis[Title/Abstract]) OR ( Pemphigus vulgaris[Title/Abstract]) OR ( Peripheral uveitis[Title/Abstract]) OR ( Pernicious anemia [Title/Abstract]) OR ( POEMS syndrome[Title/Abstract]) OR ( Polyarteritis nodosa[Title/Abstract]) OR ( Polymyalgia rheumatica[Title/Abstract]) OR ( Polymyositis[Title/Abstract]) OR ( postpericardiotomy syndrome[Title/Abstract]) OR ( Postural orthostatic tachycardia syndrome [Title/Abstract]) OR ( primary biliary cholangitis[Title/Abstract]) OR ( Primary biliary cirrhosis [Title/Abstract]) OR ( Primary sclerosing cholangitis [Title/Abstract]) OR ( Primary systemic vasculitisa [Title/Abstract]) OR ( Progressive facial hemiatrophy[Title/Abstract]) OR ( Psoriasis[Title/Abstract]) OR ( Psoriatic arthritis[Title/Abstract]) OR ( Pulmonary fibrosis[Title/Abstract]) OR ( Pure red cell aplasia [Title/Abstract]) OR ( Pyoderma gangrenosum[Title/Abstract]) OR ( Rasmussen's encephalitis[Title/Abstract]) OR ( Raynaud’s syndrome[Title/Abstract]) OR ( Reflex sympathetic dystrophy syndrome [Title/Abstract]) OR ( Reiter’s syndrome[Title/Abstract]) OR ( Relapsing polychondritis[Title/Abstract]) OR ( Restless leg syndrome [Title/Abstract]) OR ( Rheumatic fever[Title/Abstract]) OR ( Rheumatic fever and Rheumatic [Title/Abstract]) OR ( Rheumatoid arthritis[Title/Abstract]) OR ( Sarcoidosis[Title/Abstract]) OR ( Schmidt syndrome[Title/Abstract]) OR ( Scleritis[Title/Abstract]) OR ( Scleroderma [Title/Abstract]) OR ( Sclerosing Mesenteritis[Title/Abstract]) OR ( Serpiginous choroidopathy[Title/Abstract]) OR ( Sjögren’s syndrome[Title/Abstract]) OR ( Small fiber sensory neuropathy[Title/Abstract]) OR ( Stiff person syndrome [Title/Abstract]) OR ( Subacute bacterial endocarditis [Title/Abstract]) OR ( Subacute cutaneous lupus[Title/Abstract]) OR ( Susac syndrome[Title/Abstract]) OR ( Sydenham's chorea[Title/Abstract]) OR ( Sympathetic ophthalmia[Title/Abstract]) OR ( Systemic lupus erythematosus[Title/Abstract]) OR ( Systemic sclerosis[Title/Abstract]) OR ( Takayasu’s arteritis [Title/Abstract]) OR ( temporal arteritis [Title/Abstract]) OR ( Testicular autoimmunity [Title/Abstract]) OR ( Thyroiditis [Title/Abstract]) OR ( Tolosa-Hunt syndrome[Title/Abstract]) OR ( Transverse myelitis [Title/Abstract]) OR ( Tubulointerstitial nephritis uveitis syndrome [Title/Abstract]) OR ( Type 1 diabetes[Title/Abstract]) OR ( Ulcerative colitis [Title/Abstract]) OR ( Undifferentiated connective tissue disease [Title/Abstract]) OR ( Uveitis [Title/Abstract]) OR ( Vasculitis[Title/Abstract]) OR ( VEXAS Syndrome[Title/Abstract]) OR ( Vitiligo[Title/Abstract]) OR ( Vogt-Koyanagi-Harada syndrome [Title/Abstract]) OR ( Wegener’s granulomatosis[Title/Abstract]) OR ( Willis-Ekbom disease[Title/Abstract])) AND (((Pharmacokinetics[MeSH Terms]) OR (pharmacodynamic*[Title/Abstract]) OR (pharmacokinetic*[Title/Abstract]) OR (PKPD[Title/Abstract])) AND (Model*[Title/Abstract])) AND (english[Filter]) NOT (("clinical trial"[Publication Type]) OR ("clinical trial protocol"[Publication Type]) OR ("clinical trial, phase i"[Publication Type]) OR ("clinical trial, phase ii"[Publication Type]) OR ("clinical trial, phase iii"[Publication Type]) OR ("clinical trial, phase iv"[Publication Type]) OR (dog[Title/Abstract]) OR (dogs[Title/Abstract]) OR (Chagas [Title/Abstract]) OR ( "review"[Publication Type]) OR ( "systematic review"[Publication Type]) OR ( "meta analysis"[Publication Type]))

# Supplementary Tables

**Supplementary Table 1. Table of immune components incorporated into the mechanistic models.** All variables presented in the models (except for Boolean network models) were combined into 60 unified terms presented in the column headings of the table (214 variables from 36 models). A plus sign, “+”, in a cell indicates the presence of that variable in the model.

| **Reference** | **Ab** | **Ag** | **APC** | **Bcell** | **CCL20** | **CD4_Tcell** | **CD8_Tcell** | **Chl** | **Comp** | **CXCL10** | **DC** | **EASI** | **EC** | **Fib** | **GMCSF** | **HC** | **Hrmn** | **IC** | **IFNg** | **IL** | **IL1** | **IL10** | **IL12** | **IL13** | **IL15** | **IL17** | **IL2** | **IL21** | **IL22** | **IL23** | **IL31** | **IL4** | **IL6** | **IL8** | **IS** | **Inf** | **IPG** | **Lym** | **M** | **M1** | **M2** | **Neu** | **NK** | **OSC** | **OX40L** | **PL** | **SBI** | **STAT** | **Tcell** | **TD** | **Teff** | **Th** | **Th1** | **Th17** | **Th2** | **Th22** | **Tm** | **TNFa** | **Treg** | **TSLP** |
| --- | --- | --- | --- | --- | --- | --- | --- | --- | --- | --- | --- | --- | --- | --- | --- | --- | --- | --- | --- | --- | --- | --- | --- | --- | --- | --- | --- | --- | --- | --- | --- | --- | --- | --- | --- | --- | --- | --- | --- | --- | --- | --- | --- | --- | --- | --- | --- | --- | --- | --- | --- | --- | --- | --- | --- | --- | --- | --- | --- | --- |
| Yazdani et al. (1) | + | + |  |  |  |  |  |  |  |  |  |  |  |  |  |  |  | + |  |  |  |  |  |  |  |  |  |  |  |  |  |  |  |  | + | + |  |  |  |  |  |  |  |  |  |  |  |  |  | + |  |  |  |  |  |  |  |  |  |  |
| Budu-Grajdeanu et al. (2) |  |  |  |  |  |  |  |  |  |  |  |  |  |  |  |  |  | + |  |  |  |  |  |  |  |  |  |  |  |  |  |  |  |  |  | + |  |  |  |  |  |  |  |  |  |  |  |  |  | + |  |  |  |  |  |  |  |  |  |  |
| Hao et al. (3)  Karagiannis et al. (4) |  |  |  |  |  |  |  |  |  |  |  |  | + | + |  |  |  |  |  |  |  |  |  |  |  |  |  |  |  |  |  |  |  |  |  |  |  |  | + |  |  |  |  |  |  |  |  |  |  |  |  |  |  |  |  |  |  |  |  |  |
| Gao et al. (5) |  |  | + |  |  |  |  |  |  |  |  |  |  |  |  |  |  |  |  |  |  |  |  |  |  |  | + |  |  |  |  |  |  |  |  |  |  |  |  |  |  |  | + |  |  |  |  |  | + |  |  | + |  |  |  |  |  |  | + |  |
| Rullmann et al. (6) |  |  |  |  |  |  |  |  |  |  |  |  |  |  |  |  |  |  |  |  |  |  |  |  |  |  |  |  |  |  |  |  |  |  |  |  |  |  |  |  |  |  |  |  |  |  |  |  |  |  |  |  |  |  |  |  |  |  |  |  |
| Moise et al. (7) |  |  |  |  |  |  |  |  |  |  |  |  |  | + | + |  |  |  |  |  |  |  |  |  |  | + |  |  |  | + |  |  | + |  |  |  |  |  | + |  |  |  |  | + |  |  |  |  |  |  |  |  |  | + |  |  |  | + |  |  |
| Nakada et al. (8) |  |  |  |  |  |  |  |  |  |  |  |  |  |  |  |  |  |  |  |  | + |  |  |  |  | + |  |  |  |  |  |  | + |  |  | + |  |  |  |  |  |  |  |  |  |  |  |  |  |  |  |  |  |  |  |  |  | + |  |  |
| Meyer-Hermann et al. (9) |  |  |  |  |  |  |  | + |  |  |  |  |  |  |  |  | + |  |  |  |  |  |  |  |  |  |  |  |  |  |  |  |  |  | + |  |  |  |  |  |  |  |  |  |  |  |  |  |  |  |  |  |  |  |  |  |  | + |  |  |
| Wendelsdorf et al. (10) |  | + |  |  |  | + |  |  |  |  | + |  | + |  |  |  |  |  |  |  |  |  |  |  |  |  |  |  |  |  |  |  |  |  |  | + |  |  | + | + | + |  |  |  |  |  |  |  |  |  | + | + |  |  |  |  | + |  | + |  |
| Lo et al. (11,12)  Park et al. (13) |  |  |  |  |  |  |  |  |  |  |  |  |  | + |  |  |  |  | + |  |  |  |  |  |  |  | + |  |  |  |  | + |  |  |  |  |  |  |  |  |  |  |  |  |  |  |  |  |  |  |  |  | + |  |  |  |  |  | + |  |
| Dwivedi et al. (14) |  |  |  |  |  |  |  |  |  |  |  |  |  |  |  |  |  |  |  |  |  |  |  |  |  |  |  |  |  |  |  |  | + |  |  | + |  |  |  |  |  |  |  |  |  |  |  | + |  |  |  |  |  |  |  |  |  |  |  |  |
| Rogers et al. (15,16) |  |  |  |  |  |  |  |  |  |  | + |  |  |  | + |  |  |  | + |  |  | + | + |  |  | + |  |  | + | + |  | + | + | + |  | + |  |  | + | + | + | + | + |  |  |  |  |  |  |  |  | + | + | + | + |  |  | + |  |  |
| Demin et al. (17) | + | + | + |  |  |  |  |  |  |  |  |  | + |  |  |  |  |  | + |  |  |  |  |  | + |  |  | + |  |  |  |  |  |  |  |  |  | + |  |  |  |  |  |  |  |  |  |  |  |  |  | + |  |  |  |  |  |  |  |  |
| Nicholson et al. (18) |  |  | + |  |  |  |  |  |  |  |  |  |  |  |  |  |  |  |  |  |  |  |  |  |  |  |  |  |  |  |  |  |  |  |  |  |  |  |  |  |  |  |  |  |  |  |  |  | + |  | + |  |  |  |  |  | + |  |  |  |
| Moise et al. (19) |  |  |  |  |  |  | + |  |  | + |  |  |  | + | + |  |  |  | + |  |  |  |  |  |  | + |  |  |  | + |  | + | + |  |  |  |  |  | + | + | + |  |  | + |  |  |  |  |  |  |  |  | + | + | + |  |  |  | + |  |
| Vélez de Mendizábal et al. (20) |  |  |  |  |  |  |  |  |  |  |  |  |  |  |  |  |  |  |  |  |  |  |  |  |  |  |  |  |  |  |  |  |  |  |  |  |  |  |  |  |  |  |  |  |  |  |  |  |  |  | + |  |  |  |  |  |  |  | + |  |
| Kannan et al. (21) |  |  |  |  |  |  |  |  |  |  |  |  |  |  |  |  |  |  |  |  |  |  |  |  |  |  |  |  |  |  |  |  |  |  |  | + |  |  |  |  |  |  |  |  |  |  |  |  |  | + |  |  |  |  |  |  |  |  |  |  |
| Gross et al. (22) |  |  |  | + |  | + | + |  |  |  |  |  |  |  |  |  |  |  |  |  |  |  |  |  |  |  |  |  |  |  |  |  |  |  |  |  |  |  |  |  |  |  | + |  |  |  |  |  |  |  |  |  |  |  |  |  |  |  |  |  |
| Broome et al. (23) |  |  |  |  |  |  |  |  |  |  |  |  |  |  |  |  |  |  | + |  | + |  |  |  |  |  |  |  |  |  |  |  |  |  |  |  |  |  | + |  |  |  |  | + |  |  |  |  |  |  | + |  |  |  |  |  |  | + |  |  |
| Dobreva et al. (24,25) |  |  | + |  |  | + | + |  |  |  |  |  |  |  |  | + |  |  | + |  |  |  |  |  |  |  |  |  |  |  |  |  |  |  |  |  | + |  |  |  |  |  |  |  |  |  |  |  |  |  |  |  |  |  |  |  |  |  |  |  |
| Tanaka et al. (26)  Domínguez-Hüttinger et al. (27)  Christodoulides et al. (28) |  | + |  |  |  |  |  |  |  |  | + |  |  |  |  |  |  |  |  |  |  |  |  |  |  |  |  |  |  |  |  |  |  |  | + |  |  |  |  |  |  |  |  |  |  |  | + |  |  |  |  |  |  |  | + |  |  |  |  |  |
| Miyano et al (29) |  | + |  |  |  |  |  |  |  |  |  | + |  |  |  |  |  |  | + |  |  |  |  | + |  | + |  |  | + |  | + | + |  |  |  |  |  |  |  |  |  |  |  |  | + |  | + |  |  |  |  |  | + | + | + | + |  |  |  | + |
| Magombedze et al. (30) |  | + |  |  |  |  | + |  |  |  |  |  |  |  |  |  |  |  |  |  |  |  |  |  |  |  |  |  |  |  |  |  |  |  |  |  |  |  | + |  |  |  |  | + |  |  |  |  |  |  |  |  |  |  |  |  |  |  | + |  |
| Jaberi-Douraki et al. (31) |  | + |  |  |  |  |  |  |  |  |  |  |  |  |  |  |  |  |  |  |  |  |  |  |  |  | + |  |  |  |  |  |  |  |  |  |  |  |  |  |  |  |  | + |  |  |  |  |  |  | + |  |  |  |  |  |  |  | + |  |
| Salazar-Viedma et al. (32) |  | + |  |  |  |  |  |  |  |  |  |  |  |  |  |  |  |  |  |  |  |  |  |  |  |  |  |  |  |  |  |  |  |  |  |  |  |  |  |  |  |  |  | + |  |  |  |  |  |  |  |  | + | + |  |  |  |  |  |  |
| Aguda et al. (33)  Hao et al. (34) |  |  |  |  |  |  |  |  |  |  |  |  | + | + |  |  |  |  |  |  |  |  |  | + |  |  |  |  |  |  |  |  |  |  |  |  |  |  | + | + | + |  |  |  |  |  |  |  |  |  |  |  |  |  |  |  |  | + |  |  |
| Hao et al. (35) |  |  |  |  | + |  |  |  |  |  |  |  |  | + | + |  |  |  | + |  |  | + | + | + |  |  | + |  |  |  |  |  |  |  |  |  |  |  |  |  | + |  |  |  |  |  |  |  |  |  |  |  | + | + |  |  |  | + | + |  |
| van der Vegt et al. (36) |  |  |  |  |  | + |  |  |  |  |  |  |  |  |  |  |  |  |  |  |  |  |  |  |  |  |  |  |  |  |  |  |  |  | + |  |  |  |  |  |  |  |  |  |  |  |  |  |  | + |  |  |  |  |  |  |  |  | + |  |
| Head et al. (37) | + | + |  | + |  |  |  |  | + |  |  |  |  |  |  |  |  | + |  | + |  |  |  |  |  |  |  |  |  |  |  |  |  |  |  |  |  |  |  |  |  |  |  |  |  | + |  |  |  |  |  | + |  |  |  |  |  |  |  |  |
| Arazi et al. (38) |  | + |  | + |  |  |  |  |  |  |  |  |  |  |  |  |  | + |  |  |  |  |  |  |  |  |  |  |  |  |  |  |  |  |  |  |  |  |  |  |  |  |  |  |  |  |  |  |  |  |  |  |  |  |  |  |  |  |  |  |
| **Reference** | **Ab** | **Ag** | **APC** | **Bcell** | **CCL20** | **CD4_Tcell** | **CD8_Tcell** | **Chl** | **Comp** | **CXCL10** | **DC** | **EASI** | **EC** | **Fib** | **GMCSF** | **HC** | **Hrmn** | **IC** | **IFNg** | **IL** | **IL1** | **IL10** | **IL12** | **IL13** | **IL15** | **IL17** | **IL2** | **IL21** | **IL22** | **IL23** | **IL31** | **IL4** | **IL6** | **IL8** | **IS** | **Inf** | **IPG** | **Lym** | **M** | **M1** | **M2** | **Neu** | **NK** | **OSC** | **OX40L** | **PL** | **SBI** | **STAT** | **Tcell** | **TD** | **Teff** | **Th** | **Th1** | **Th17** | **Th2** | **Th22** | **Tm** | **TNFa** | **Treg** | **TSLP** |
| Iwami et al. (39) |  | + |  |  |  |  |  |  |  |  |  |  |  |  |  |  |  |  |  |  |  |  |  |  |  |  |  |  |  |  |  |  |  |  |  |  |  |  |  |  |  |  |  |  |  |  |  |  |  | + |  |  |  |  |  |  |  |  |  |  |
| Ramos et al. (40) |  |  | + |  |  |  |  |  |  |  |  |  |  |  |  |  |  |  |  |  |  |  |  |  |  |  |  |  |  |  |  |  |  |  |  |  |  |  |  |  |  |  |  |  |  |  |  |  |  |  | + |  |  |  |  |  |  |  | + |  |
| Khailaie et al. (41) |  |  |  |  |  |  |  |  |  |  |  |  |  |  |  |  |  |  |  |  |  |  |  |  |  |  | + |  |  |  |  |  |  |  |  |  |  |  |  |  |  |  |  |  |  |  |  |  | + |  |  | + |  |  |  |  |  |  | + |  |
| Louzoun et al. (42) |  |  |  |  |  |  |  |  |  |  |  |  |  |  |  |  |  |  |  |  |  |  |  |  |  |  |  |  |  |  |  |  |  |  |  |  |  |  | + |  |  |  |  |  |  |  |  |  | + |  |  |  | + |  | + |  |  |  |  |  |
| Hara et al. (43) |  | + |  |  |  |  |  |  |  |  |  |  |  |  |  |  |  |  |  |  |  |  |  |  |  |  |  |  |  |  |  |  |  |  |  |  |  |  |  |  |  |  |  |  |  |  |  |  |  |  |  | + |  |  |  |  | + |  |  |  |
| Valeyev et al. (44) |  |  |  |  |  |  |  |  |  |  |  |  |  |  |  |  |  |  |  |  |  |  |  |  |  |  |  |  |  |  |  |  |  |  |  |  |  |  |  |  |  |  |  |  |  |  |  |  |  |  |  |  |  |  |  |  |  |  |  |  |

Abbreviation: Ab — Antibody; Ag — Antigen; APC — Antigen presenting cells; Bcell — B-cells; CCL20 — Chemokine (C-C motif) ligand 20; CD4_Tcell — CD4+ T-cells; CD8_Tcell — CD8+ T-cells; Chl — Cholesterol; Comp — Complement system; CXCL10 — CXCL-10; DC — Dendritic cells; EASI — Eczema Area and Severity Index; EC — Epithelial cells; Fib — Fibrosis; GMCSF — GM-CSF; HC — Hair cycle; Hrmn — Hormones; IC — Immune complexes; IFNg — IFN-γ; IL — Interleukin; IL1 — IL-1; IL10 — IL-10; IL12 — IL-12; IL13 — IL-13; IL15 — IL-15; IL17 — IL-17; IL2 — IL-2; IL21 — IL-21; IL22 — IL-22; IL23 — IL-23; IL31 — IL-31; IL4 — IL-4; IL6 — IL-6; IL8 — IL-8; IS — Immune system; Inf — Inflammation; IPG — Immune privilege guardians; Lym — Lymphocytes; M — Macrophage; M1 — M1 macrophage; M2 — M2 macrophage; Neu — Neutrophils; NK — Natural killer cells; OSC — Organ-specific cells; OX40L — OX40 ligand; PL — Plasma cells; SBI — Skin barrier integrity; STAT — Signal transducer and activator of transcription protein; Tcell — T-cells; TD — Tissue damage; Teff — Effector T-cells; Th — T-helpers; Th1 — T-helper 1; Th17 — T-helper 17; Th2 — T-helper 2; Th22 — T-helper 22; Tm — Memory T-cells; TNFa — TNF-α; Treg — Regulatory T-cells; TSLP — Thymic stromal lymphopoietin

**References:**

1. Yazdani A, Bahrami F, Pourgholaminejad A, Moghadasali R. A biological and a mathematical model of SLE treated by mesenchymal stem cells covering all the stages of the disease. *Theory Biosci Theor Den Biowissenschaften* (2023) 142:167–179. doi: 10.1007/s12064-023-00390-4

2. Budu-Grajdeanu P, Schugart RC, Friedman A, Birmingham DJ, Rovin BH. Mathematical framework for human SLE Nephritis: disease dynamics and urine biomarkers. *Theor Biol Med Model* (2010) 7: doi: 10.1186/1742-4682-7-14

3. Hao W, Rovin BH, Friedman A. Mathematical model of renal interstitial fibrosis. *Proc Natl Acad Sci* (2014) 111:14193–14198. doi: 10.1073/pnas.1413970111

4. Karagiannis G, Hao W, Lin G. Calibrations and validations of biological models with an application on the renal fibrosis. *Int J Numer Methods Biomed Eng* (2020) 36:e3329. doi: 10.1002/cnm.3329

5. Gao X, He J, Sun X, Li F. Dynamically modeling the effective range of IL-2 dosage in the treatment of systemic lupus erythematosus. *iScience* (2022) 25:104911. doi: 10.1016/j.isci.2022.104911

6. Rullmann J a. C, Struemper H, Defranoux NA, Ramanujan S, Meeuwisse CML, van Elsas A. Systems biology for battling rheumatoid arthritis: application of the Entelos PhysioLab platform. *Syst Biol* (2005) 152:256–262. doi: 10.1049/ip-syb:20050053

7. Moise N, Friedman A. Rheumatoid arthritis - a mathematical model. *J Theor Biol* (2019) 461:17–33. doi: 10.1016/j.jtbi.2018.10.039

8. Nakada T, Mager DE. Systems model identifies baseline cytokine concentrations as potential predictors of rheumatoid arthritis inflammatory response to biologics. *Br J Pharmacol* (2022) 179:4063–4077. doi: 10.1111/bph.15845

9. Meyer-Hermann M, Figge MT, Straub RH. Mathematical modeling of the circadian rhythm of key neuroendocrine-immune system players in rheumatoid arthritis: a systems biology approach. *Arthritis Rheum* (2009) 60:2585–2594. doi: 10.1002/art.24797

10. Wendelsdorf K, Bassaganya-Riera J, Hontecillas R, Eubank S. Model of colonic inflammation: immune modulatory mechanisms in inflammatory bowel disease. *J Theor Biol* (2010) 264:1225–1239. doi: 10.1016/j.jtbi.2010.03.027

11. Lo W-C, Arsenescu RI, Friedman A. Mathematical model of the roles of T cells in inflammatory bowel disease. *Bull Math Biol* (2013) 75:1417–1433. doi: 10.1007/s11538-013-9853-2

12. Lo W-C, Arsenescu V, Arsenescu RI, Friedman A. Inflammatory Bowel Disease: How Effective Is TNF-α Suppression? *PloS One* (2016) 11:e0165782. doi: 10.1371/journal.pone.0165782

13. Park A, Kim S, Jung IH, Byun JH. An immune therapy model for effective treatment on inflammatory bowel disease. *PloS One* (2020) 15:e0238918. doi: 10.1371/journal.pone.0238918

14. Dwivedi G, Fitz L, Hegen M, Martin SW, Harrold J, Heatherington A, Li C. A multiscale model of interleukin-6-mediated immune regulation in Crohn’s disease and its application in drug discovery and development. *CPT Pharmacomet Syst Pharmacol* (2014) 3:e89. doi: 10.1038/psp.2013.64

15. Rogers KV, Martin SW, Bhattacharya I, Singh RSP, Nayak S. A Dynamic Quantitative Systems Pharmacology Model of Inflammatory Bowel Disease: Part 1 - Model Framework. *Clin Transl Sci* (2021) 14:239–248. doi: 10.1111/cts.12849

16. Rogers KV, Martin SW, Bhattacharya I, Singh RSP, Nayak S. A Dynamic Quantitative Systems Pharmacology Model of Inflammatory Bowel Disease: Part 2 - Application to Current Therapies in Crohn’s Disease. *Clin Transl Sci* (2021) 14:249–259. doi: 10.1111/cts.12850

17. Demin OO, Smirnov SV, Sokolov VV, Cucurull-Sanchez L, Pichardo-Almarza C, Flores MV, Benson N, Demin OV. Modeling of celiac disease immune response and the therapeutic effect of potential drugs. *BMC Syst Biol* (2013) 7:56. doi: 10.1186/1752-0509-7-56

18. Nicholson D, Kerr EC, Jepps OG, Nicholson LB. Modelling experimental uveitis: barrier effects in autoimmune disease. *Inflamm Res Off J Eur Histamine Res Soc Al* (2012) 61:759–773. doi: 10.1007/s00011-012-0469-z

19. Moise N, Friedman A. A mathematical model of the multiple sclerosis plaque. *J Theor Biol* (2021) 512:110532. doi: 10.1016/j.jtbi.2020.110532

20. Vélez de Mendizábal N, Carneiro J, Solé RV, Goñi J, Bragard J, Martinez-Forero I, Martinez-Pasamar S, Sepulcre J, Torrealdea J, Bagnato F, et al. Modeling the effector - regulatory T cell cross-regulation reveals the intrinsic character of relapses in Multiple Sclerosis. *BMC Syst Biol* (2011) 5:114. doi: 10.1186/1752-0509-5-114

21. Kannan V, Kiani NA, Piehl F, Tegner J. A minimal unified model of disease trajectories captures hallmarks of multiple sclerosis. *Math Biosci* (2017) 289:1–8. doi: 10.1016/j.mbs.2017.03.006

22. Gross CC, Pawlitzki M, Schulte-Mecklenbeck A, Rolfes L, Ruck T, Hundehege P, Wiendl H, Herty M, Meuth SG. Generation of a Model to Predict Differentiation and Migration of Lymphocyte Subsets under Homeostatic and CNS Autoinflammatory Conditions. *Int J Mol Sci* (2020) 21:2046. doi: 10.3390/ijms21062046

23. Broome TM, Coleman RA. A mathematical model of cell death in multiple sclerosis. *J Neurosci Methods* (2011) 201:420–425. doi: 10.1016/j.jneumeth.2011.08.008

24. Dobreva A, Paus R, Cogan NG. Mathematical model for alopecia areata. *J Theor Biol* (2015) 380:332–345. doi: 10.1016/j.jtbi.2015.05.033

25. Dobreva A, Paus R, Cogan NG. Analysing the dynamics of a model for alopecia areata as an autoimmune disorder of hair follicle cycling. *Math Med Biol J IMA* (2018) 35:387–407. doi: 10.1093/imammb/dqx009

26. Tanaka G, Domínguez-Hüttinger E, Christodoulides P, Aihara K, Tanaka RJ. Bifurcation analysis of a mathematical model of atopic dermatitis to determine patient-specific effects of treatments on dynamic phenotypes. *J Theor Biol* (2018) 448:66–79. doi: 10.1016/j.jtbi.2018.04.002

27. Domínguez-Hüttinger E, Christodoulides P, Miyauchi K, Irvine AD, Okada-Hatakeyama M, Kubo M, Tanaka RJ. Mathematical modeling of atopic dermatitis reveals “double-switch” mechanisms underlying 4 common disease phenotypes. *J Allergy Clin Immunol* (2017) 139:1861-1872.e7. doi: 10.1016/j.jaci.2016.10.026

28. Christodoulides P, Hirata Y, Domínguez-Hüttinger E, Danby SG, Cork MJ, Williams HC, Aihara K, Tanaka RJ. Computational design of treatment strategies for proactive therapy on atopic dermatitis using optimal control theory. *Philos Transact A Math Phys Eng Sci* (2017) 375:20160285. doi: 10.1098/rsta.2016.0285

29. Miyano T, Irvine AD, Tanaka RJ. A mathematical model to identify optimal combinations of drug targets for dupilumab poor responders in atopic dermatitis. *Allergy* (2022) 77:582–594. doi: 10.1111/all.14870

30. Magombedze G, Nduru P, Bhunu CP, Mushayabasa S. Mathematical modelling of immune regulation of type 1 diabetes. *Biosystems* (2010) 102:88–98. doi: 10.1016/j.biosystems.2010.07.018

31. Jaberi-Douraki M, Pietropaolo M, Khadra A. Continuum model of T-cell avidity: Understanding autoreactive and regulatory T-cell responses in type 1 diabetes. *J Theor Biol* (2015) 383:93–105. doi: 10.1016/j.jtbi.2015.07.032

32. Salazar-Viedma M, Vergaño-Salazar JG, Pastenes L, D’Afonseca V. Simulation Model for Hashimoto Autoimmune Thyroiditis Disease. *Endocrinology* (2021) 162:bqab190. doi: 10.1210/endocr/bqab190

33. Aguda BD, Marsh CB, Thacker M, Crouser ED. An in silico modeling approach to understanding the dynamics of sarcoidosis. *PloS One* (2011) 6:e19544. doi: 10.1371/journal.pone.0019544

34. Hao W, Crouser ED, Friedman A. Mathematical model of sarcoidosis. *Proc Natl Acad Sci U S A* (2014) 111:16065–16070. doi: 10.1073/pnas.1417789111

35. Hao W, Marsh C, Friedman A. A Mathematical Model of Idiopathic Pulmonary Fibrosis. *PloS One* (2015) 10:e0135097. doi: 10.1371/journal.pone.0135097

36. van der Vegt SA, Polonchuk L, Wang K, Waters SL, Baker RE. Mathematical modelling of autoimmune myocarditis and the effects of immune checkpoint inhibitors. *J Theor Biol* (2022) 537:111002. doi: 10.1016/j.jtbi.2021.111002

37. Head M, Meryhew N, Runquist O. Mechanism and computer simulation of immune complex formation, opsonization, and clearance. *J Lab Clin Med* (1996) 128:61–74. doi: 10.1016/s0022-2143(96)90114-6

38. Arazi A, Neumann AU. Modeling immune complex-mediated autoimmune inflammation. *J Theor Biol* (2010) 267:426–436. doi: 10.1016/j.jtbi.2010.08.033

39. Iwami S, Takeuchi Y, Miura Y, Sasaki T, Kajiwara T. Dynamical properties of autoimmune disease models: tolerance, flare-up, dormancy. *J Theor Biol* (2007) 246:646–659. doi: 10.1016/j.jtbi.2007.01.020

40. Ramos MPM, Ribeiro C, Soares AJ. A kinetic model of T cell autoreactivity in autoimmune diseases. *J Math Biol* (2019) 79:2005–2031. doi: 10.1007/s00285-019-01418-4

41. Khailaie S, Bahrami F, Janahmadi M, Milanez-Almeida P, Huehn J, Meyer-Hermann M. A mathematical model of immune activation with a unified self-nonself concept. *Front Immunol* (2013) 4:474. doi: 10.3389/fimmu.2013.00474

42. Louzoun Y, Atlan H, Cohen IR. Modeling the influence of TH1- and TH2-type cells in autoimmune diseases. *J Autoimmun* (2001) 17:311–321. doi: 10.1006/jaut.2001.0548

43. Hara A, Iwasa Y. Autoimmune diseases initiated by pathogen infection: Mathematical modeling. *J Theor Biol* (2020) 498:110296. doi: 10.1016/j.jtbi.2020.110296

44. Valeyev NV, Hundhausen C, Umezawa Y, Kotov NV, Williams G, Clop A, Ainali C, Ouzounis C, Tsoka S, Nestle FO. A systems model for immune cell interactions unravels the mechanism of inflammation in human skin. *PLoS Comput Biol* (2010) 6:e1001024. doi: 10.1371/journal.pcbi.1001024
